# Full-Length Galectin-3 Is Required for High Affinity Microbial Interactions and Antimicrobial Activity

**DOI:** 10.3389/fmicb.2021.731026

**Published:** 2021-10-08

**Authors:** Shang-Chuen Wu, Alex D. Ho, Nourine A. Kamili, Jianmei Wang, Kaleb L. Murdock, Richard D. Cummings, Connie M. Arthur, Sean R. Stowell

**Affiliations:** ^1^Joint Program in Transfusion Medicine, Department of Pathology, Brigham and Women’s Hospital, Harvard Medical School, Boston, MA, United States; ^2^Center for Transfusion Medicine and Cellular Therapies, Emory University School of Medicine, Atlanta, GA, United States; ^3^Department of Surgery, Beth Israel Deaconess Medical Center, Harvard Medical School, Boston, MA, United States

**Keywords:** galectin, blood group, microbe, antimicrobial, molecular mimicry

## Abstract

While adaptive immunity enables the recognition of a wide range of microbial antigens, immunological tolerance limits reactively toward self to reduce autoimmunity. Some bacteria decorate themselves with self-like antigens as a form of molecular mimicry to limit recognition by adaptive immunity. Recent studies suggest that galectin-4 (Gal-4) and galectin-8 (Gal-8) may provide a unique form of innate immunity against molecular mimicry by specifically targeting microbes that decorate themselves in self-like antigens. However, the binding specificity and antimicrobial activity of many human galectins remain incompletely explored. In this study, we defined the binding specificity of galectin-3 (Gal-3), the first galectin shown to engage microbial glycans. Gal-3 exhibited high binding toward mammalian blood group A, B, and αGal antigens in a glycan microarray format. In the absence of the N-terminal domain, the C-terminal domain of Gal-3 (Gal-3C) alone exhibited a similar overall binding pattern, but failed to display the same level of binding for glycans over a range of concentrations. Similar to the recognition of mammalian glycans, Gal-3 and Gal-3C also specifically engaged distinct microbial glycans isolated and printed in a microarray format, with Gal-3 exhibiting higher binding at lower concentrations toward microbial glycans than Gal-3C. Importantly, Gal-3 and Gal-3C interactions on the microbial microarray accurately predicted actual interactions toward intact microbes, with Gal-3 and Gal-3C displaying carbohydrate-dependent binding toward distinct strains of *Providentia alcalifaciens* and *Klebsiella pneumoniae* that express mammalian-like antigens, while failing to recognize similar strains that express unrelated antigens. While both Gal-3 and Gal-3C recognized specific strains of *P. alcalifaciens* and *K. pneumoniae*, only Gal-3 was able to exhibit antimicrobial activity even when evaluated at higher concentrations. These results demonstrate that while Gal-3 and Gal-3C specifically engage distinct mammalian and microbial glycans, Gal-3C alone does not possess antimicrobial activity.

## Introduction

Galectins are an ancient and evolutionarily conserved protein family that have a diverse range of functions relevant to a wide variety of diseases (Liu and Rabinovich, 2005, 2010; Vasta, 2009). Among carbohydrate binding proteins, galectins are the most widely expressed in all organisms and primarily engage counter ligands through recognition of β-galactose-containing glycoconjugates (Barondes et al., 1994). Galectins have been classified into three major groups based on their quaternary structural features, prototypical, tandem repeat, and chimeric (Liu and Rabinovich, 2010; Arthur et al., 2015a). Among these, Gal-3 is the only chimeric galectin, possessing a single carbohydrate recognition domain (CRD) and a self-aggregating N-terminal domain rich in proline, glycine, and tyrosine residues which can mediate oligomerization in presence of multivalent ligands (Hsu et al., 1992; Ahmad et al., 2004a; Morris et al., 2004).

In addition to modulating host cell function through engagement of cell surface carbohydrates, galectins can also interact directly with bacterial surface glycans (Vasta, 2009). Gal-3 in particular was the first shown to engage bacterial glycans where early studies demonstrated binding to lipopolysaccharides (LPS) isolated from *Pseudomonas aeruginosa*, *Klebsiella pneumoniae*, *Neisseria gonorrhoeae*, *Neisseria meningitidis,* and *Helicobacter pylori* (Mey et al., 1996; Gupta et al., 1997; John et al., 2002; Fowler et al., 2006; Quattroni et al., 2012; Vasta, 2012). Although interactions between Gal-3 and the LPS of *N. meningitidis* in particular appear to involve carbohydrate recognition through its C-terminal CRD domain (Vinogradov and Perry, 2001), the fine specificity of Gal-3 for microbial glycans, many of which can be quite diverse and distinct in structure, remains incompletely understood.

The ability of galectins to engage bacterial glycans may represent an important element of host immunity. While adaptive immunity can target a nearly infinite range of antigens, the breadth of this ability is tempered by tolerance mechanisms that limit reactivity toward self. Although this may reduce the probability of autoimmunity, this creates a gap in adaptive immunity toward microbes that decorate themselves in self-like antigens as a form of molecular mimicry (Arthur et al., 2015c). Previous studies demonstrated that Gal-4 and Gal-8 in particular can kill strains of *Escherichia coli* through recognition of bacterial surface glycans that mimic blood group antigens (Stowell et al., 2010). However, despite early studies demonstrating that Gal-3 can bind LPS (Mey et al., 1996; Gupta et al., 1997; John et al., 2002; Fowler et al., 2006; Quattroni et al., 2012), the overall antimicrobial activity of Gal-3, including key features of the quaternary structure of Gal-3 responsible for this antimicrobial activity, remains relatively unexplored.

As carbohydrate recognition have been previously shown to reside within the C-terminal domain of Gal-3 (Seetharaman et al., 1998), in this study, we examined the binding specificity of Gal-3 and Gal-3C over a range of concentrations using a series of glycan microarrays populated with mammalian or microbial glycans. While Gal-3 and Gal-3C possess similar overall binding specificity, full-length Gal-3 was required for higher affinity binding toward glycans on each array, suggesting that oligomerization status through the N-terminal domain likely plays a key role in higher affinity glycan recognition. Importantly, the relative affinity of Gal-3 toward glycans on the microbial glycan microarray (MGM) accurately predicted actual antimicrobial activity. However, while Gal-3 and Gal-3C both engaged microbial glycans and intact microbes, only Gal-3 possessed microbicidal activity.

## Materials and Methods

### Protein Expression and Purification of Human Gal-3 by *Escherichia coli*

Expression plasmids encoding human Gal-3 and Gal-3C were transformed into *E.coli* BL21 (DE3), and Gal-3 and Gal-3C were then expressed as outlined previously (Stowell et al., 2010; Wu et al., 2021b). Briefly, transformed bacteria were cultured in LB broth containing 100μg/ml ampicillin with agitation (250rpm) at 37°C. When bacteria were grown to the mid-log phase, protein expression was induced by addition of isopropyl 1-thio-β-D-galactopyranoside (IPTG, 1.5mM). After 20-h induction in 16°C, 6L cultured bacteria were pelleted and harvested by centrifugation and then resuspended in 60ml bacterial lysis buffer (PBS with 14mM 2-mercaptoethanol (2-ME), 60μl ribonuclease A (RNase A), 60μl DNase I, 60μl lysozyme, and 2 protease inhibitor cocktail tablets). The suspension was passed through a cell disruptor, and the lysate was centrifuged at 17,000rpm at 4°C for 1h. Supernatant was applied to lactosyl-sepharose affinity chromatography column. For elution, the elution buffer (PBS with 14mM 2-ME and 100mM lactose) was added. The desired fractions were pooled and stained with Coomassie blue on SDS-PAGE gel to test purity (Supplementary Figure 1). Before derivatization, 2-ME and lactose were removed from Gal-3 using a PD-10 gel filtration column for bacteria killing assay.

### Effect of Recombinant Gal-3 on Bacteria Viability

When assaying potential antimicrobial effects of Gal-3 and Gal-3C, each strain was assessed in the mid-logarithmic growth phase (OD_600_ of ~0.1) and grown in LB media as outlined previously (Arthur et al., 2015b). Bacterial cells were incubated with the concentrations of each galectin indicated in the figure legends (0.04–10μM) at 37°C for 2h with shaking at 250rpm. Bacteria were then pipetted and plated on LB agar plate to determine the number of viable bacteria by CFU enumeration.

### Bacterial Strains

*Providentia alcalifaciens* O5 and *P. alcalifaciens* O21 were kindly provided by Y. Knirel (ND Zelinsky Institute of Organic Chemistry, Moscow, Russia). *K. pneumoniae* O1 and *K. pneumoniae* O4 were kindly provided by C. Whitfield (University of Guelph). Each bacteria strain was grown and maintained at 37°C using LB culture medium (Fisher).

### Mammalian Glycan Array Analysis

Galectins were labeled with Alexa Fluor^™^ 488 NHS Ester (succinimidyl ester) by incubating 2mg/ml galectin with 1mg Alexa Fluor^™^ 488 for 1h at room temperature and avoid from light as outlined previously (Stowell et al., 2004). Unconjugated Alexa Fluor^™^ 488 and free lactose were separated using a PD-10 gel filtration column (GE Healthcare). Labeled galectin was purified again by lactosyl-sepharose column to remove any inactive protein generated during the labeling process. Bound galectin was eluted with 100mM lactose in PBS plus 2-ME. While 2-ME is not required for Gal-3 activity, this approach was used to provide a consistent protocol for all galectin purification. Importantly, 2-ME and lactose were then removed using PD-10 gel filtration column. Finally, labeled galectin was applied to CFG glycan microarray (CFG) and MGM prepared as described previously (Blixt et al., 2004; Stowell et al., 2008a, 2014; Song et al., 2009; Wu et al., 2021a). For galectin recognition of glycans on the printed glycan microarray, the slides were blocked with blocking buffer (500mg of BSA in 50ml PBST) for 1h at room temperature. Slides were then incubated with directly labeled Gal-3 or Gal-3C at the indicated concentrations using binding buffer (500mg of BSA in 50ml PBST with 14mM 2-ME) for 1h at room temperature in a dark humid chamber. As noted previously, while 2-ME is not required for Gal-3 stability, as it is required to maintain the activity of other galectins, we have employed this binding buffer for all galectin assays to provide a uniform approach when assessing galectin binding specificity using glycan microarrays. Slides were then washed by successive immersion in PBST containing 0.5% Tween 20 (four times), PBS (four times), and H_2_O (four times). The slide was dried by microcentrifugation, and an image of bound fluorescence was obtained using a microarray scanner (GenePix 4000 B, Molecular devices). Integrated spot intensities were obtained using Imagene software (GenePix Pro 7). The heat map was created by GraphPad Prism 8 (Prism 8) as outlined previously (Verkerke et al., 2021), which was also used to ascertain dissociation constants (*K_D_*). For non-saturated positive glycan interactions, the relative fluorescence units were plotted as a percent of the maximal binding at the highest concentration examined.

### Flow Cytometry Analysis

To examine potential binding by each galectin, bacteria were resuspended and washed twice in PBS at 4°C and then incubated with 0.1μM Alexa Fluor^™^ 488 labeled Gal-3 or Gal-3C at 4°C for 20min. In some experiments, Gal-3 or Gal-3C were co-incubated with 20mM thiodigalactoside (TDG) for 10min before incubation with the bacteria as a control. After incubation, cells were washed twice and resuspended them in 400μl PBS for flow cytometry analysis using FACSCanto II flow cytometer (BD Biosciences). The data were processed with FlowJo version 10.

## Results

### Gal-3 and Gal-3C Display Similar Preferences for Blood Group Antigens

**Figure 1:**
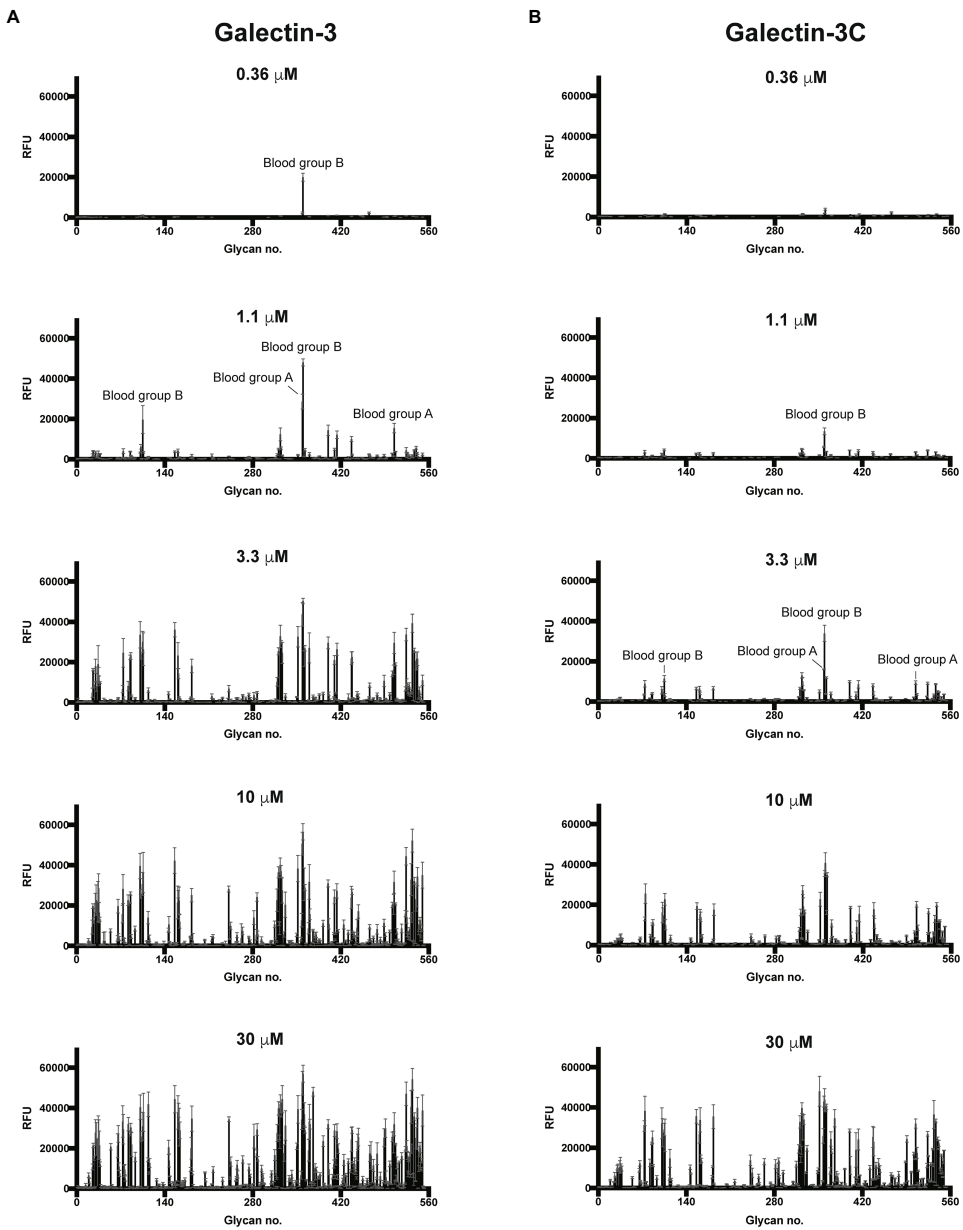
Gal-3 and Gal-3C preferentially recognize blood group antigens at distinct concentrations. Consortium for functional glycomics (CFG) glycan microarray data obtained after incubation with the indicated concentrations of Gal-3 **(A)** and Gal-3C **(B)**. RFU, relative fluorescence units. Error bars represent means ± standard deviation (SD).

To better understand the binding specificity and affinity of Gal-3, including the influence of the N-terminal domain, for glycan ligands, we first examined its binding specificity using the Consortium for Functional Glycomics (CFG) glycan microarray. To accomplish this, we expressed the Gal-3 and Gal-3C (Supplementary Figure 1), followed by the evaluation of both proteins in parallel on the CFG array. As the overall apparent specificity of carbohydrate binding proteins can be influenced by the protein concentration used for array analysis and as the N-terminal domain may enhance overall binding affinity through cross linking of bound glycans, we examined glycan recognition over a range of concentrations as opposed to a single concentration primarily employed in our previous studies using glycan microarray analysis (Stowell et al., 2010). Using this approach, we found that virtually no binding could be detected for either Gal-3 or Gal-3C at or below concentrations of 0.12μM (data not shown). However, at 0.36μM, recognition of blood group B was observed by Gal-3, but not by Gal-3C (Figures 1A,B). Nevertheless, at 1.1μM, binding toward the same blood group B antigen was observed for Gal-3C (Figure 1B). Binding toward additional glycan ligands, primarily polymorphic blood group antigens, became readily apparent following incubation of Gal-3 at higher concentration. However, in contrast to Gal-3, incubation with 3.3μM Gal-3C was required to achieve a similar level of absolute binding toward the same initial blood group B antigen bound by Gal-3 at 0.36μM (Figure 1B). Similar to Gal-3, at higher concentrations, additional glycan recognition, including a strong preference for blood group antigens, could be detected for Gal-3C, which generally mirrored glycan recognition by Gal-3 at lower concentrations (Figures 1A,B).

**Figure 2:**
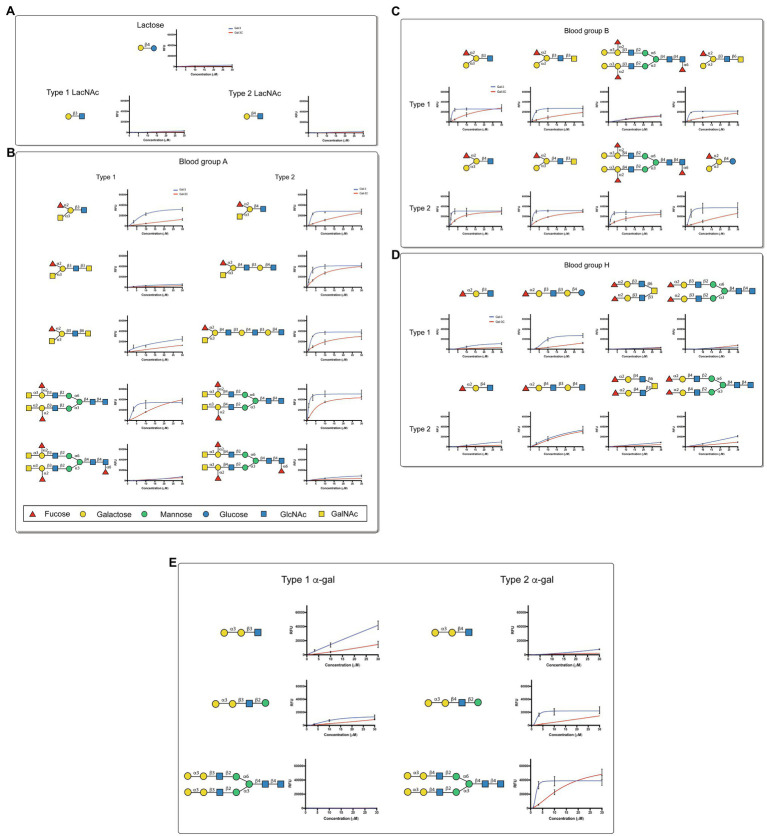
Gal-3 displays high affinity for blood group antigens on a CFG glycan microarray. (A-E) Binding isotherms generated following incubation of Gal-3 (blue) and Gal- 3C (red) on the CFG glycan microarray are shown for lactose and N-acetyllactosamine (LacNAc) (A), blood group A containing glycan structures (B), blood group B containing glycan structures (C), H antigen (blood group O) containing glycan structures (D) and -Gal containing glycan structures (E). Detailed Symbol Nomenclature for Glycans (SNFG) structures are shown. Error bars represent means ± standard deviation (SD). GalNAc: *N*-Acetylgalactosamine; GlcNAc: *N*-Acetylglucosamine.

Although overall binding specificity of Gal-3 and Gal-3C appeared to display high level of similarity when adjusted for concentration, the apparent affinity of each protein for individual glycan ligands differed. In order to define the relatively affinity of Gal-3 and Gal-3C for glycan ligands on the CFG microarray in more detail, we next examined binding isotherms generated following incubation of each galectin over a range of concentrations. Given the high affinity interactions observed toward blood group antigens, we specifically evaluated Gal-3 and Gal-3C binding toward distinct blood group antigen types as presented on the CFG array. While very little binding could be observed toward lactose (Galβ1-4Glc) or type 1 or type 2 LacNAc (Galβ1-3GlcNAc or Galβ1-4GlcNAc, respectively; Figure 2A), similar high affinity interactions were observed for blood group A and blood group B, although Gal-3 and Gal-3C each displayed a slightly higher affinity for type 2 blood group A and blood group B antigens than type 1 antigens (Figures 2B,C). In contrast, Gal-3 and Gal-3C appeared to possess a lower affinity for the H antigens regardless of type 1 or type 2 configuration when compared to blood group A or B (Figure 2D). However, the fucose modification present in B antigens likely positively influences Gal-3 and Gal-3C blood group recognition, as neither Gal-3 nor Gal-3C displayed similar binding affinity toward αGal containing type 1 or type 2 structures despite the fact that these glycans terminate in the blood group B disaccharide (Figure 2E). These results suggest that Gal-3 has high affinity for blood group antigens and that both terminal glycan modifications (α1-2Fuc and α1-3GalNAc or Gal) present in blood group A and blood group B are likely required to support higher affinity interactions by Gal-3.

**Figure 3:**
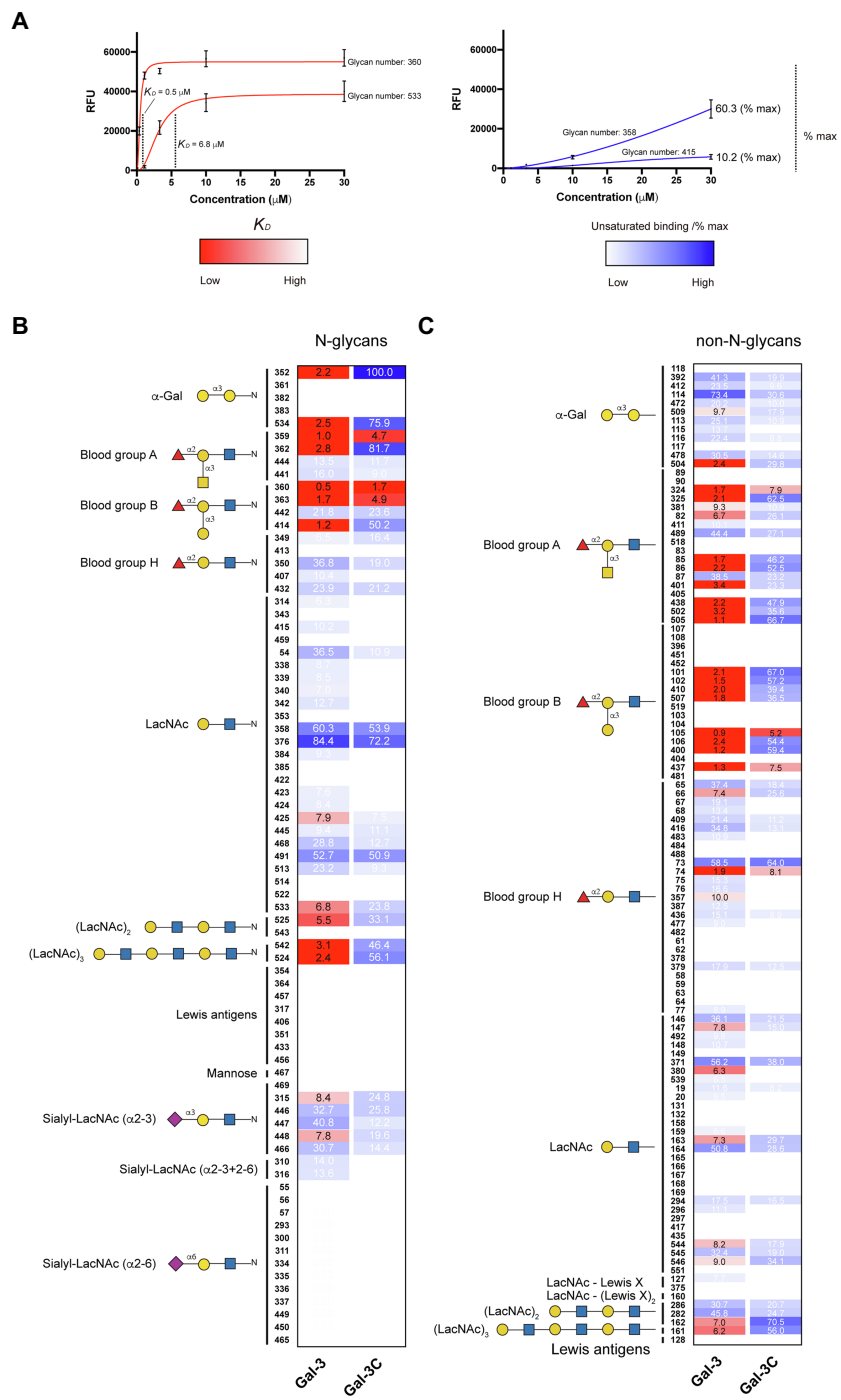
*K_D_* values for Gal-3 and Gal-3C binding toward blood group antigens and other mammalian glycans. **(A)** Representative binding isotherms used to generate *K_D_* values and the % max of the highest concentration tested for unsaturated glycans. **(B,C)** Selected blood group antigens were shown along with heat map representation of *K_D_* values (red) and the % max of the highest concentration tested for unsaturated glycans (blue) for antigens located on N-glycans **(B)** and non-N-glycans **(C)**. The heat map from darker red (low *K_D_*) to light red (high *K_D_*) is shown. For the unsaturated binding, the heat map from light blue (low % max) to darker blue (high % max) is shown. Examples of glycans examined are annotated to the left of each heat map as structures that are present on N-glycans (N-glycans) as shown in **(B)** or as the isolated glycan motifs (non-N-glycans) as shown in **(C)**.

In order to compare the relative binding affinities of Gal-3 and Gal-3C toward blood group antigens and other glycan structures present on the array, we calculated the relative *K_D_* generated from binding isotherm data for each galectin toward various blood group antigens, polylactosamine (polyLacNAc), and other common structural modifications previously shown to influence galectin recognition (e.g., α2-6 sialylation). As there are many distinct glycan determinants in this array format, we highlighted *K_D_* values for general classes of glycans; the detailed structural information for each glycan shown is available in supplemental data (Supplementary Table 1). As the binding profile against some glycans did not saturate over the concentrations employed in this analysis (Figure 1), we compared the relatively weak, but detectable binding observed toward glycans where saturation did not occur as a percentage of the maximal binding on the array at the highest concentration examined (30 μM). This was done to capture binding that did occur, but that failed to saturate over the concentrations tested. Using this approach, relative differences in weaker binding profiles could be highlighted while clearly separating these binding profiles from higher binding interactions where saturation did occur, and therefore, relative *K_D_* values could be ascertained (Figure 3A). Using this approach, blood group antigens are clearly some of the highest affinity ligands for Gal-3, although polyLacNAc structures, such as (LacNAc)_2_ and (LacNAc)_3_, were also bound with high affinity as well (Figure 3B). In contrast, appreciably *K_D_* values for Gal-3C were only apparent for blood group antigens over the concentrations examined, while binding toward polyLacNAc glycans was certainly detected at the higher concentrations (Figure 3C).

### Gal-3 and Gal-3C Bind to Microbial Glycans Decorated With Mammalian-Like Structures

**Figure 4:**
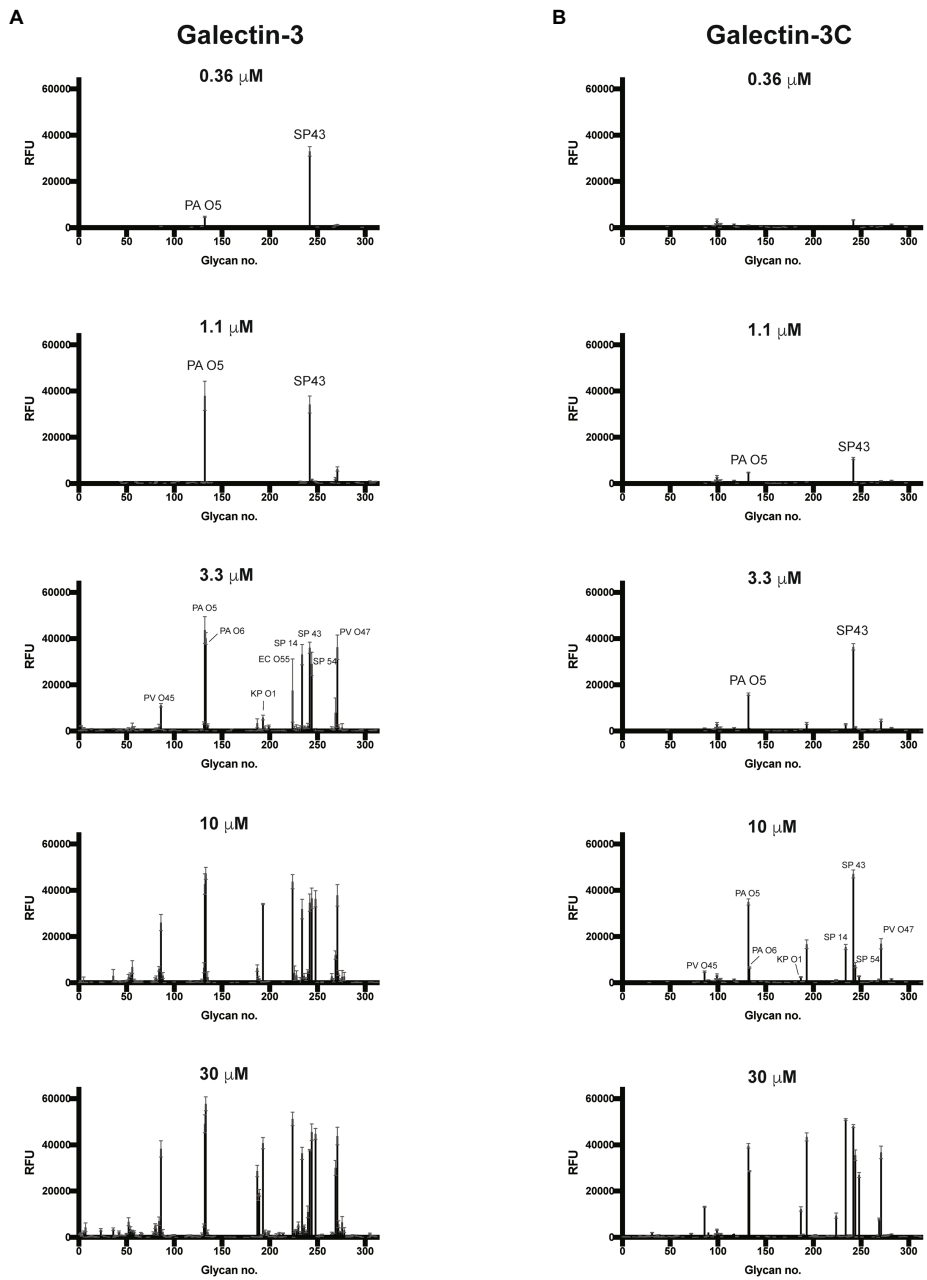
Gal-3 and Gal-3C recognize distinct microbial glycans **(A,B)** Microbial glycan microarray (MGM) data obtained after incubation with the Gal-3 **(A)** or Gal-3C **(B)** at the concentrations indicated. Error bars represent means ± standard deviation (SD). RFU, relative fluorescence units; PA O5, *Providencia alcalifaciens* O5; PA O6, *P. alcalifaciens* O6; KP O1, *Klebsiella pneumoniae* O1; SP 14, *Streptococcus pneumoniae* type 14; SP 43, *S. pneumoniae* type 43; SP54, *S. pneumoniae* type 54; PV O45, *Proteus vulgaris* O45; PV O47, *P. vulgaris* O47; EC O55, *Escherichia coli* O55.

Given the proclivity of Gal-3 and Gal-3C for blood group antigens on the CFG arrays, we next sought to determine whether the same level of specificity occurs when similar ligands are instead presented on a microbial glycan. To accomplish this, we examined Gal-3 and Gal-3C binding toward more than 300 microbial glycans isolated from distinct strains of bacteria using a previously characterized MGM (Supplementary Table 2; Stowell et al., 2014; Wesener et al., 2015). Similar to binding on the CFG array, appreciable Gal-3 binding was not detected until a concentration of 0.36μM Gal-3 was employed (Figure 4A). The structure recognized at this concentration was the glycan isolated from *Streptococcus pneumoniae* 43. When examining Gal-3 glycan recognition at a slightly higher concentration of 1.1μM, the O antigen of *Providentia alcalifaciens* O5 was also recognized. At higher concentrations, the glycan antigens of additional microbes were detected, including the O antigen of *Klebsiella pneumoniae* O1. In contrast to Gal-3, detectable binding on the MGM was not observed for Gal-3C until at least 1.1μM was employed, with appreciable binding toward *S. pneumoniae* type 43 or *P. alcalifaciens* O5 only apparent following incubation with 3.3μM Gal-3C (Figure 4B). However, similar to results obtained following incubation with the CFG array, while differences in the concentrations needed to detect binding were certainly apparent between Gal-3 and Gal-3C, the overall trends in bindings specificity were similar, strongly suggesting that while the N-terminal domain likely facilitates higher affinity binding, the intrinsic specificity for individual glycans appears to be driven by the C-terminal domain.

**Figure 5:**
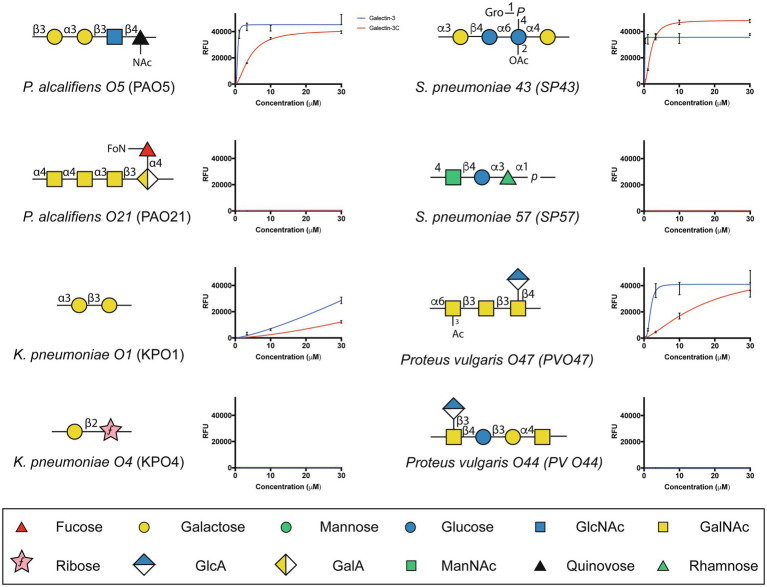
Gal-3 and Gal-3C exhibit high affinity interactions with select microbial glycans. The binding isotherms of Gal-3 (blue) and Gal-3C (red) for microbial glycans are shown. The structure for each corresponding glycan is depicted on the left of each binding isotherm. Symbols used to represent each monosaccharide present in each bacterial glycan are represented in the legend below. Error bars represent means ± standard deviation (SD). GlcA: D-Glucuronic acid, GalA: D-Galacturonic acid and ManNAc: N-Acetyl-D-mannosamine

**Figure 6:**
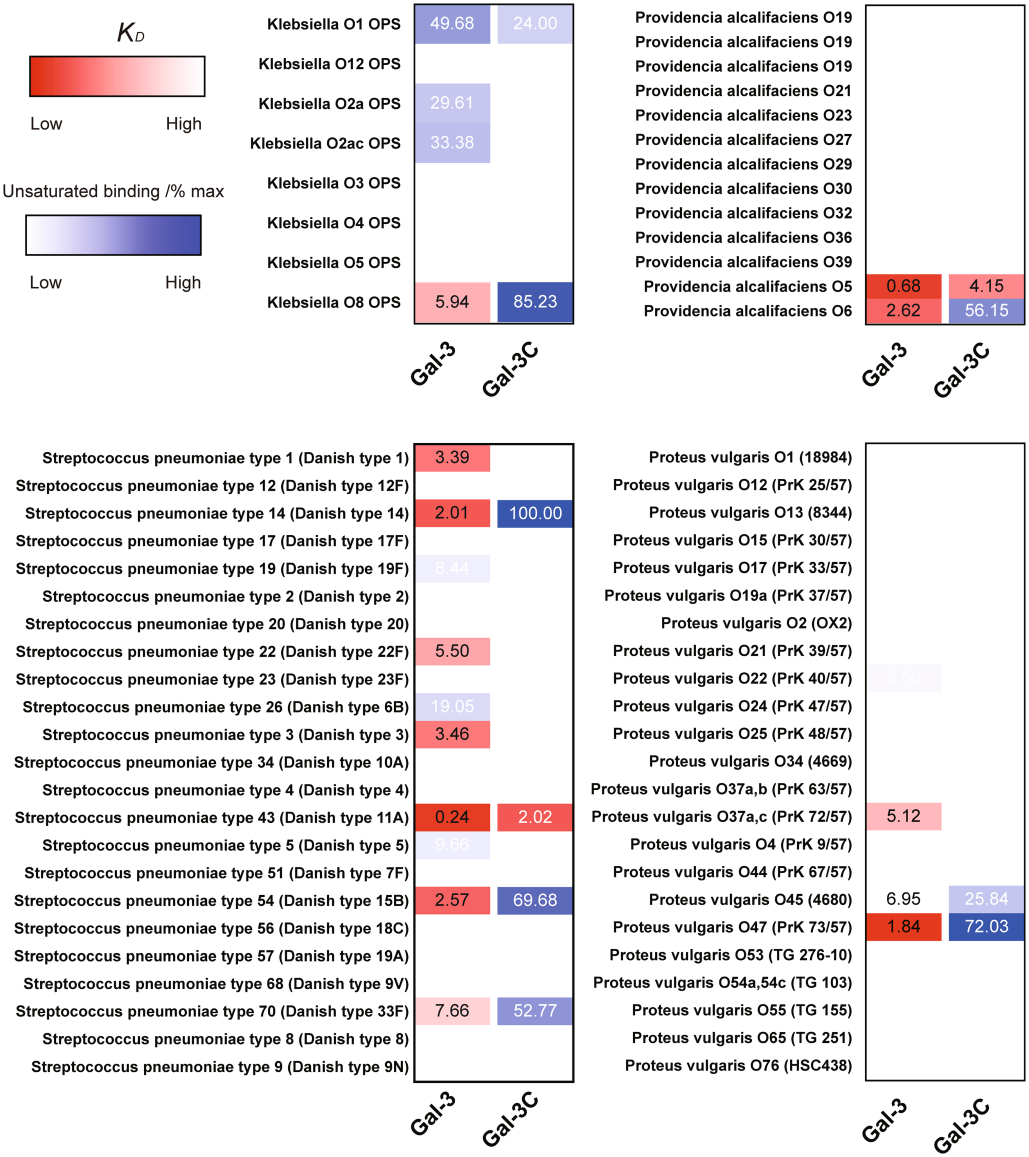
*K_D_* values of Gal-3 and Gal-3C binding to microbial glycans. Selected bacteria are presented along with heat map representation of *K_D_* and unsaturated binding (% max). The *K_D_* and % max values were sorted by red and blue color, respectively. The heat map from darker red (low *K_D_*) to light red (high *K_D_*). For the unsaturated binding, the heat map from light blue (low % max) to darker blue (high % max).

The ability of Gal-3 and Gal-3C to bind the isolated glycans from *S. pneumoniae* type 43 or *P. alcalifaciens* O5 at concentrations similar to that observed on the CFG array was intriguing in part because the intrinsic structure of each glycan reflects lactose and αGal antigens, respectively (Figure 5); Gal-3 exhibited low binding toward these individual structures on the CFG array (Figures 2A,E). As a result, we next explored in more detail the binding affinity of Gal-3 and Gal-3C toward the microbial glycans present on the MGM using the same approach outlined for evaluating saturated and non-saturated binding toward the CFG arrays. Using this approach, we observed a very high apparent affinity for the glycan antigens of *S. pneumoniae* type 43 or *P. alcalifaciens* O5, with relatively *K_D_* values of 0.24 and 0.68μM, respectively (Figure 6). In contrast, binding to the glycan of *K. pneumoniae* O1 by Gal-3 was apparent, but much weaker, where binding failed to fully saturate and therefore provide a relative *K_D_* over the concentrations tested (Figure 6). Importantly, Gal-3 and Gal-3C binding did not appear to reflect indiscriminate engagement of microbial glycans, as neither exhibited appreciable binding toward related strains of microbes, such as *P. alcalifaciens* O21, *Streptococcus pneumoniae* 57, or *K. pneumoniae* O4, which fail to express glycan with mammalian-like structural motifs (Figure 5). These results suggest that while Gal-3 can certainly recognize microbial glycans, this recognition exhibits a certain level of specificity, with most microbial glycans not recognized by Gal-3 or Gal-3C.

### Gal-3, but Not Gal-3C, Kills *P. alcalifaciens* O5 and *K. pneumoniae* O1

**Figure 7:**
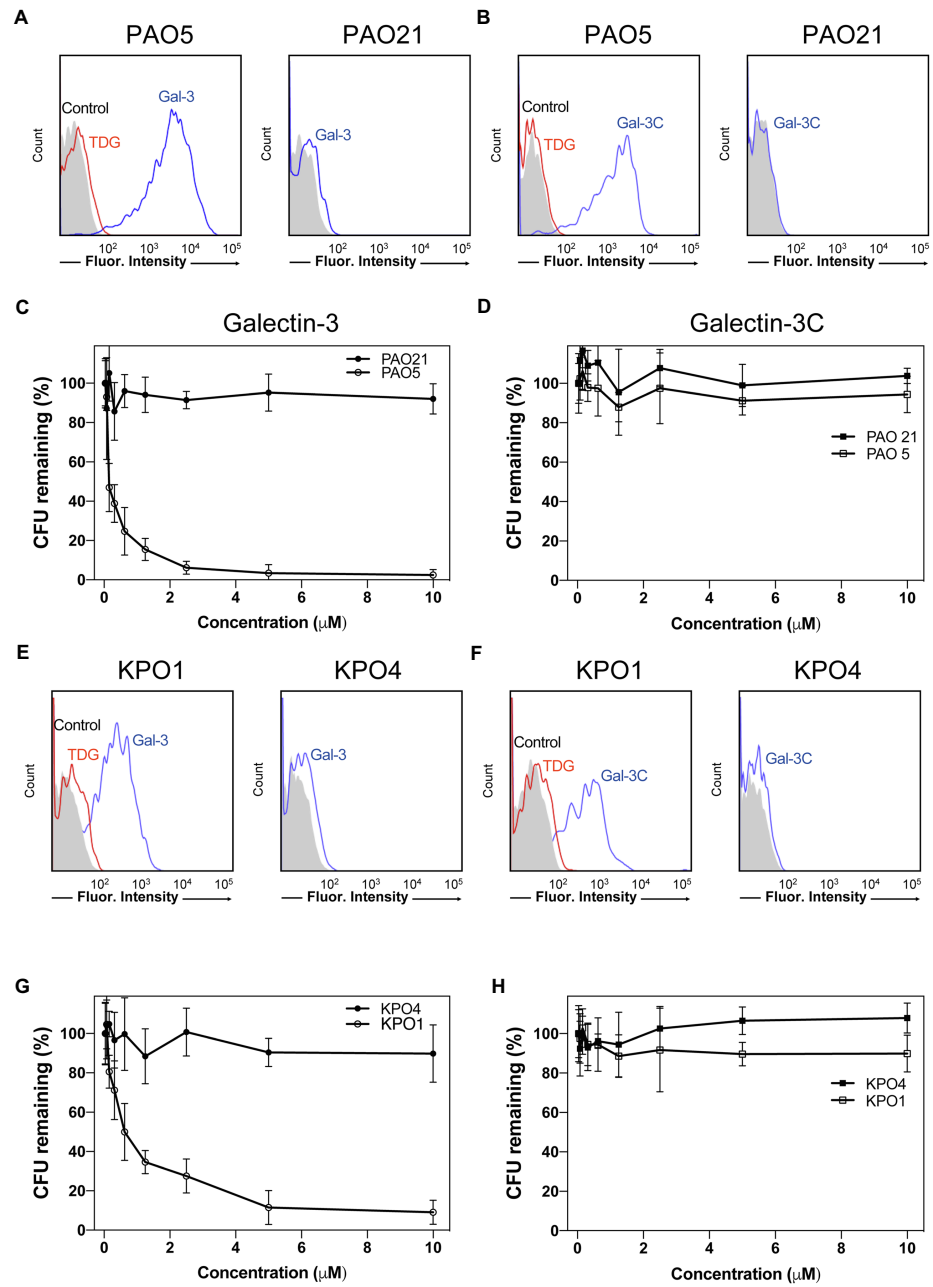
Gal-3 and Gal-3C recognize and kill *Providencia alcalifaciens* O5 (PA O5) and *Klebsiella pneumoniae* O1 (KP O1). **(A,B)** Flow cytometric analysis of Gal-3 **(A)** and Gal-3C **(B)** binding to PA O5 and PA O21 with or without inclusion of 20mM thiodigalactoside (TDG) as indicated. **(C,D)** Quantification of viable bacteria after incubation with the indicated concentrations Gal-3 **(C)** and Gal-3C **(D)**. **(E,F)** Flow cytometric analysis of Gal-3 **(E)** and Gal-3C **(F)** binding to KP O1 and KP O4 with or without inclusion of 20mM TDG as indicated. **(G,H)** Quantification of viable bacteria after incubation with the indicated concentrations Gal-3 **(G)** and Gal-3C **(H)**. Error bars represent means ± SD.

Given the ability of Gal-3 to differentially recognize microbial glycans on the MGM, we next sought to determine whether binding on the microarray accurately predicted actual interactions and overall antimicrobial potency toward intact microbes. Clear interactions between Gal-3 or Gal-3C and *P. alcalifaciens* O5 could be observed by flow cytometric examination (Figures 7A,B). Engagement of *P. alcalifaciens* O5 by both Gal-3 and Gal-3C also required carbohydrate recognition, as inclusion of TDG, a non-metabolizable inhibitor of galectin-glycan interactions, inhibited recognition (Figures 7A,B). Recognition by Gal-3 and Gal-3C appeared to be specific to *P. alcalifaciens* O5 as incubation with *P. alcalifaciens* O21 failed to result in any detectable binding when evaluated in parallel (Figures 7A,B). To determine the impact of Gal-3 and Gal-3C engagement of *P. alcalifaciens* O5 on microbial viability, we next examined the outcome of Gal-3 or Gal-3C incubation with *P. alcalifaciens* O5 over a range of concentrations. Incubation of Gal-3 resulted in reduced viability of *P. alcalifaciens* O5, with an effective concentration 50 (EC50) of around 0.17μM. In contrast, incubation with the same concentrations of Gal-3 with *P. alcalifaciens* O21 failed to result in any detectable impact on microbial viability (Figure 7C). To determine whether Gal-3C can likewise impact microbial viability, we incubated *P. alcalifaciens* O5 with Gal-3C. Unlike Gal-3, Gal-3C failed to influence the viability of *P. alcalifaciens* O5 at all concentrations tested; similar results were observed following incubation of Gal-3C with *P. alcalifaciens* O21 (Figure 7D).

While Gal-3 and Gal-3C recognized a variety of glycan determinants isolated from distinct strains of microbes, the apparent affinity differed, suggesting that differential killing activity may also occur toward distinct microbial targets. Furthermore, whether the inability of Gal-3C to kill *P. alcalifaciens* O5 is limited to this strain of microbe remained unknown. As a result, we next evaluated the binding of Gal-3 and Gal-3C toward *K. pneumoniae* O1 as both Gal-3 and Gal-3C displayed detectable, albeit lower, binding toward the O antigen isolated from this microbe (Figure 6). Similar to Gal-3 and Gal-3C interactions with *P. alcalifaciens* O5, Gal-3 and Gal-3C not only bound *K. pneumoniae* O1, but these interactions likewise depended on carbohydrate recognition as inclusion of TDG prevented binding. Engagement of *K. pneumoniae* O1 also appeared to be specific, as similar binding failed to occur when evaluated against *K. pneumoniae* O4 (Figures 7E,F). To determine the sensitivity of *K. pneumoniae* O1 to Gal-3, we next evaluated *K. pneumoniae* O1 viability following incubation with increasing concentrations of Gal-3. While loss of microbial viability was noted at higher concentrations, the EC50 of Gal-3 toward *K. pneumoniae* O1 was higher (0.75μM), suggesting that like binding, killing activity toward *K. pneumoniae* O1 required higher concentrations of Gal-3. Also similar to binding, Gal-3 failed to impact the viability of *K. pneumoniae* O4 at all concentrations tested (Figure 7G). To determine whether Gal-3C possesses the ability to kill *K. pneumoniae* O1, we next examined *K. pneumoniae* O1 viability following incubation with Gal-3C. Similar to its inability to impact the viability of *P. alcalifaciens* O5, Gal-3C likewise failed to impact the viability of *K. pneumoniae* O1 or *K. pneumoniae* O4 (Figure 7H). Taken together, these results suggest that the MGM can ascertain relative affinity and microbicidal potency of Gal-3 toward distinct strains of microbes and that the N-terminal domain is required for both high affinity interactions with microbial glycans and the overall antimicrobial activity of Gal-3.

## Discussion

While galectins have long been recognized as carbohydrate binding proteins defined by their ability to engage β-galactose-containing glycans, the fine specificity of many galectins, especially toward microbial glycans, has remained incompletely defined. Gal-3 in particular is intriguing as it is the only galectin that belongs to the chimeric type subfamily, where it possesses a unique N-terminal domain that does not possess critical residues responsible for carbohydrate recognition, but is required for oligomerization (Hsu et al., 1992; Chiu et al., 2020; Zhao et al., 2021). While many studies have examined the binding specificity of the full-length protein (Hirabayashi et al., 2002; Stowell et al., 2008a; Song et al., 2009; Horlacher et al., 2010; Gao et al., 2019), much less is known regarding the intrinsic specificity of Gal-3C toward a wide variety of glycan determinants. The results of the present study suggest that the specificity of Gal-3 for most glycans appears to reside within the C-terminal domain with higher affinity interactions with glycan ligands requiring the full-length protein. The present results also demonstrate that full-length Gal-3 is required for its antimicrobial activity.

Although general binding toward β-galactose containing glycans became a defining feature of galectins, modifications of β-galactose can have a significant impact on overall glycan recognition. The preference for ABO(H) glycans has become an intriguing and almost defining feature of galectins (Feizi et al., 1994; Hirabayashi et al., 2002; Carlsson et al., 2007; Stowell et al., 2008a; Arthur et al., 2015d; Kamili et al., 2016), although the extent to which other galectins likewise possess similar overall binding preferences remains to be defined. The overall binding preferences exhibited by Gal-3 in the present study are largely in agreement with earlier studies, where Gal-3 was observed to exhibit higher binding to blood group A and blood group B than the H antigen (Feizi et al., 1994). More recent studies suggested that galectins may exhibit a slight preference for blood group B over blood group A (Stowell et al., 2010). Consistent with this, microarray analysis in the present study demonstrated that blood group B was the first glycan bound at the lowest concentration of Gal-3 or Gal-3C examined for which any appreciable glycan recognition could be observed. At slightly higher concentrations, binding to blood group A could also be readily detected. However, analysis of galectin binding at a single concentration on the microarray can be misleading, as such an approach only ascertains relative binding at a given concentration without providing the additional insight obtained when examining galectin binding over a range of concentrations that allows the establishment a binding isotherm capable of providing relative *K_D_* values. Using this approach, the impact of subtle differences in blood group presentation can become more apparent.

The selective forces that facilitated ABO(H) blood group polymorphism evolution within the human population have remained incompletely understood (Cooling, 2015; Stowell and Stowell, 2019a,b). However, several studies suggest that various pathogens may have influenced the selection of ABO(H) polymorphisms (Reid and Bird, 1990; Cooling, 2015), much like other blood group and blood group-like antigens that can likewise be a barrier to blood transfusion and the optimal use of similar therapeutics (Zerra et al., 2017, 2021; Mener et al., 2018; Patel et al., 2018; Arthur et al., 2021). The polymorphic nature of ABO(H) antigens strongly suggests that the high binding affinity of Gal-3 toward these antigens is not due to selective pressures that facilitate the engagement and modulation of host cells. Rather, this preference points to an evolutionary process that likely selected for this binding specificity in the context of host immunity toward microbes. In this way, galectins may provide a unique form of innate immunity against microbes that utilize molecular mimicry to avoid adaptive immunity. As innate immune factors are not subjected to tolerogenic programs that limit adaptive immunity toward self-antigens, galectins and perhaps other lectins may fill this gap in adaptive immunity by targeting microbes that express mammalian-like structures on their surface.

The ability of Gal-3 to recognize a diverse range of microbes that express distinct self-like antigens is intriguing and suggests that the relatively promiscuous binding profile often attributed to this protein family over a range of concentrations may actually reflect an important ability to recognize a variety of microbial glycans with self-like antigen features. However, there are clearly differences in the binding that can be observed toward microbial glycans and similar motifs as presented on mammalian glycans. For example, while Gal-3 failed to exhibit a high level of binding toward lactose, presentation of this motif within the microbial glycan of *S. pneumoniae* type 43 appeared to support high affinity glycans. These results strongly suggest that the presentation of a given glycan motif, possibly due to the polymerizing nature of repeating structures on the microbial surface, may be important glycan feature that facilitates this type of interaction. Consistent with this, while almost no detectable binding was observed for Gal-3 toward LacNAc, polymers of LacNAc in the form of polyLacNAc supported high affinity Gal-3 interactions. However, subtle differences in glycan presentation on the microbial surface can still impact overall Gal-3 binding. For example, while *K. pneumoniae* O1 and *P. alcalifaciens* O5 contain the Galα1-3Gal motif, this structure is polymerized within distinct glycans on each microbe (Galα1-3Galβ1-3Gal-R in *K. pneumoniae* O1 and Galα1-3Galβ1-3GlcNAc-R in *P. alcalifaciens* O5). Unfortunately, a major limitation in the MGM is the lack of availability of most of the microbes represented on the array. While this limited the ability to perform confirmatory tests for all positive events observed, the correlation between binding and the potency of killing activity toward *K. pneumoniae* O1 and *P. alcalifaciens* O5 suggests that this overall approach may be useful when seeking to examine the binding specificity of a given carbohydrate binding protein for microbial glycans. Despite subtle differences in binding affinity unique microbial glycans, Gal-3 and Gal-3C displayed a fairly high level of specificity for distinct microbial glycans when compared to all the microbial glycans present on the array. This stands in stark contrast to most innate immune factors that often recognize microbial motifs that are common to a diverse range of microbes (Janeway and Medzhitov, 2002). This unique specificity for individual strains of microbes places galectins as unique innate immune factors that selectively bind and kill a subset of microbes.

Glycan microarrays have become a powerful way to examine the binding specificity of carbohydrate binding proteins against a wide range of glycan determinants (Rillahan and Paulson, 2011). The construction of microarrays requires less glycan material than many other assay formats and therefore expands the ability to explore a particular glycan library when assessing the binding specificity of a given carbohydrate binding protein. While microarray approaches for assessing carbohydrate binding proteins have improved the overall analysis of carbohydrate binding proteins specificity, the manufacturing and use of glycan microarrays can remain resource intense, and therefore, analysis has primarily focused on a single concentration of a particular carbohydrate binding protein on a given microarray. This approach can uncover important features of glycan binding for a particular carbohydrate binding protein, including glycan modifications that appear to directly inhibit glycan recognition. However, when using this approach, it can be challenging to know *a priori* where the linear range of glycan binding for a particular carbohydrate binding protein resides. Similarly, while the density of glycans printed is relatively similar to discrete glycans, printing can result in subtle differences in glycan concentration that can impact the maximal binding possible for a given carbohydrate binding protein. While these differences can be subtle, they can suggest possible differences in glycan binding affinity that may actually reflect slight differences in printing efficiency between different glycans. The ability to examine Gal-3 and Gal-3C binding over a range of concentrations provided a relative binding affinity that may aid in reducing variability due to slight differences in glycan printing density, while also providing a general framework for assessing the actual affinity for a given glycan as printed in an array format. Using this approach, a number of glycans were bound at higher concentration where saturation was not achieved but where binding was clearly detected. To document these lower affinity interactions, we employed the more commonly ranked analysis as a percentage of maximal binding only at the highest concentration tested. This combined approach of *K_D_* analysis for glycans that clearly saturated coupled with a relative binding assessment of unsaturated glycans builds on recent advances in glycan array analysis with the goal of providing additional insight into carbohydrate binding protein glycan recognition. More definitive *K_D_* values could have been obtained for lower affinity interaction by expanding the concentrations tested. However, as galectin concentrations in excess of 30μM *in vivo* are unlikely, the relevance of *K_D_* ascertained following escalating test concentrations beyond 30μM is of uncertain value, and therefore, analysis was limited to the concentration range tested.

A variety of previous studies has examined the requirement of the N-terminal domain in Gal-3 signaling of host cells, with a primary focus on immune cells (Chen et al., 2005). Through N-terminal domain self-association, Gal-3 can cross link counter receptors and impact the signaling outcomes of many host cells (Horlacher et al., 2010; Guha et al., 2013; Gao et al., 2019). However, less has been known regarding the involvement for the N-terminal domain in Gal-3-mediated antimicrobial activity and overall binding to a wide range of both mammalian and microbial glycans. Given the similarities in overall specificity, despite significant differences in the concentration at which binding was detected on each array, the intrinsic affinity of glycans within the Gal-3 CRD may not differ whether in the context of the full-length protein or as an isolated CRD. Consistent with this, several studies using solution-based isothermal calorimetry demonstrated that Gal-3 and Gal-3C exhibit very similar affinity for various glycan ligands (Ahmad et al., 2004b; Rodriguez et al., 2015). Given the ability of the N-terminal domain to facilitate Gal-3 self-association (Hsu et al., 1992; Chiu et al., 2020; Zhao et al., 2021), initial binding by one CRD within the full-length protein may increase the effective concentration of the second CRD toward glycans immobilized on the same surface, directly increasing the probability that additional binding events occur in the context of the multimeric protein. In this context, the microscopic *K_a_* or binding affinity of each domain within the full-length protein is likely no different than the CRD alone, but the impact of enhanced effective concentration of each CRD within the oligomeric full-length protein following initial binding likely increases the overall avidity of interactions with immobilized glycans; this can be observed as an apparent increase in affinity for mammalian and microbial glycans. Recent studies have demonstrated that the C-terminal domain of Gal-3 can also self-associate (Lepur et al., 2012; Sundqvist et al., 2018), suggesting that higher order Gal-3 structures may form independent of the N-terminal domain. However, while the C-terminal domain may self-associate following engagement of microbial glycans or on the microbial surface in general, this interaction does not appear to be sufficient to convey antimicrobial activity following Gal-3C binding.

Prior studies defining the antimicrobial activity of galectins have primarily focused on the tandem repeat galectins, Gal-4 and Gal-8, which possess two distinct CRDs linked by an intervening peptide (Levy et al., 2001; Rustiguel et al., 2016). Examination of the components of Gal-8 in particular that are required for killing microbes demonstrated that the C-terminal domain (Gal-8C) alone possesses its antimicrobial activity (Stowell et al., 2010). As prior data suggest that Gal-8C is a monomer (Stowell et al., 2008b), the intrinsic ability of Gal-8C to kill microbes suggested that monovalent galectin interactions with microbial glycans alone can alter microbial viability. Given these prior findings, we anticipated that Gal-3C, despite lacking its intrinsic ability to oligomerize, may likewise possess the ability to kill microbes. In contrast, despite being able to engage blood group antigens with similar affinity as Gal-8C (Stowell et al., 2008b), Gal-3C failed to impact microbial viability. It is possible that the requirement of the N-terminal domain for Gal-3-mediated microbial killing reflects an activity that is completely independent of its role in facilitating multimerization. However, as inclusion of hapten inhibitors prevented Gal-3 microbial binding and killing, initial engagement of microbes likely requires recognition of glycan ligands by the Gal-3 CRD. Consistent with this, Gal-3 also failed to recognize or kill microbes that do not express self-like antigens. These results do not rule out the possibility that the N-terminal domain may facilitate Gal-3 interactions with the microbial surface following initial engagement by Gal-3. Examination of the N-terminal domain alone will be required to determine whether this isolated domain possesses the ability to directly interact with microbes. As Gal-4 and Gal-8 do not possess a similar N-terminal domain as Gal-3, yet possess the ability to effectively kill microbes, the requirement of full-length Gal-3 for microbial killing may indeed reflect a need for N-terminal domain-mediated oligomerization. Since oligomerization status is crucial for Gal-3 to mediate many carbohydrate-dependent processes (Horlacher et al., 2010; Gao et al., 2019), proteolytic cleavage on the N-terminal domain may reflect a regulatory circuit that modulates its antimicrobial activity among other regulatory features of the protein (Hsu et al., 1992; Herrmann et al., 1993).

The outcome of Gal-3 binding to bacterial glycans may not be limited to antimicrobial killing. Gal-3 can facilitate LPS detection by neutrophils and directly impact neutrophil activation (Li et al., 2008; Fermino et al., 2011), suggesting that Gal-3 may not only serve as a danger-associated molecular pattern molecule (Sato and Nieminen, 2002), but may also alter the ability of innate immune cells and perhaps other cells, to detect pathogen associated molecular patterns. Some of these interactions may be mediated by direct interactions with lipid A (Mey et al., 1996). However, the present results suggest that the composition of the glycan present on LPS may influence these interactions and attendant consequences. Indeed, the ability of Gal-3 to engage specific microbial glycan determinants may not only play a role in providing direct protection against molecular mimicry, but also may have related consequences on the ability of Gal-3 to detect LPS shed from individual strains of microbes and therefore alert or otherwise alter a host immune response following exposure to a given microbe.

Taken together, these results demonstrate that Gal-3 binds a very diverse range of mammalian glycans, but exhibits a high affinity for polymorphic blood group antigens, a process that appears to require the full-length protein. However, whether Gal-3 can successfully bind and kill similar microbes *in vivo* remains to be tested. Despite the ability of the C-terminal domain of Gal-8 alone to kill bacteria, Gal-3C fails to alter microbial viability, suggesting that some self-association of Gal-3 that occurs independent of the C-terminal domain alone is likely required for the ability of Gal-3 to kill microbes. These results also demonstrate that Gal-3 binding alone is not sufficient to kill bacteria, as examination of Gal-3C at concentrations that achieved similar levels of microbial glycan binding as was observed by Gal-3 failed to kill bacteria. Thus, the N-terminal domain of Gal-3 is required not only for high affinity microbial glycan interactions, but also for the ability of Gal-3 to kill microbes.

## Data Availability Statement

The original contributions presented in the study are included in the article/Supplementary Material, and further inquiries can be directed to the corresponding authors.

## Author Contributions

S-CW, CA, and SS conceived the project, which was facilitated by AH, NK, JW, KM, and RC who provided critical reagents, experimental support, and critical discussion. S-CW and SS wrote the manuscript, which was additionally commented on and edited by the remaining authors. All authors contributed to the article and approved the submitted version.

## Funding

This work was supported in part by the Burroughs Wellcome Trust Career Award for Medical Scientists, the National Institutes of Health Early Independence grant DP5OD019892, and UO1 CA242109 to SS.

## Conflict of Interest

The authors declare that the research was conducted in the absence of any commercial or financial relationships that could be construed as a potential conflict of interest.

## Publisher’s Note

All claims expressed in this article are solely those of the authors and do not necessarily represent those of their affiliated organizations, or those of the publisher, the editors and the reviewers. Any product that may be evaluated in this article, or claim that may be made by its manufacturer, is not guaranteed or endorsed by the publisher.
