# Supplementary material for: Full-Length Galectin-3 Is Required for High Affinity Microbial Interactions and Antimicrobial Activity
Source: Front Microbiol. 2021 Oct 8;12:731026. doi: 10.3389/fmicb.2021.731026 (PMC8531552; doi:10.3389/fmicb.2021.731026)
Supplement: Supplementary file 1 [file Data_Sheet_1.PDF]

Wu et al. Supplementary Figure 1.

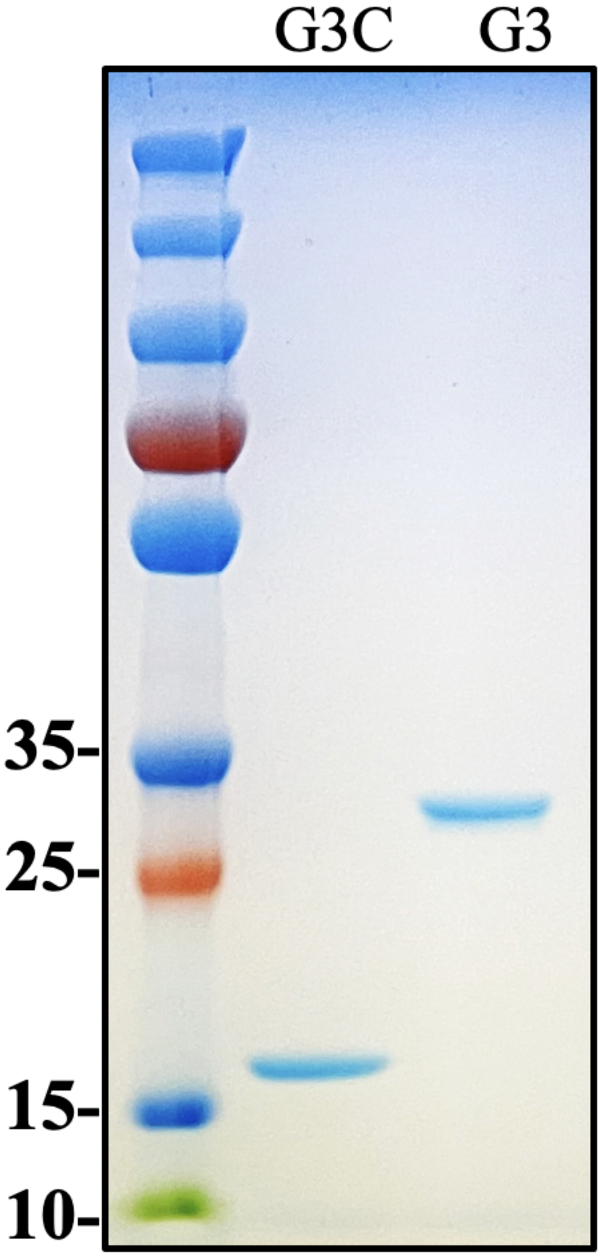

Supplementary Figure 1. Human Gal-3 and Gal-3C were purified by lactosyl-sepharose column chromatography. Each protein was subjected to SDS-PAGE and stained with Coomassie Brilliant Blue. Molecular weight for each marker is given in kDa.
