# Supplementary material for: Full-Length Galectin-3 Is Required for High Affinity Microbial Interactions and Antimicrobial Activity
Source: Front Microbiol. 2021 Oct 8;12:731026. doi: 10.3389/fmicb.2021.731026 (PMC8531552; doi:10.3389/fmicb.2021.731026)
Supplement: Supplementary file 2 [file Data_Sheet_2.PDF]

Wu et al. Supplementary Figure 2.

K_D: Low — High

Unsaturated binding /% max: Low — High

	Gal-3	Gal-3C
1-2 Mannan Acetobacter methanolieus MB135		
Davanat		
Escherichia coli K13		
Escherichia coli K235 LPS		
Escherichia coli K5		
Escherichia coli K92		
Escherichia coli O106		
Escherichia coli O107		
Escherichia coli O111:B4		
Escherichia coli O112ab		
Escherichia coli O118		
Escherichia coli O125		
Escherichia coli O127:B8		
Escherichia coli O128-B12 LPS		
Escherichia coli O130		
Escherichia coli O145		
Escherichia coli O148		
Escherichia coli O150		
Escherichia coli O151		
Escherichia coli O168		
Escherichia coli O180	4.91	
Escherichia coli O26:B6		
Escherichia coli O29		
Escherichia coli O40		
Escherichia coli O49		
Escherichia coli O52		
Escherichia coli O55:B5	4.46	18.51
Escherichia coli O58		
Escherichia coli O61		
Escherichia coli O71		
Escherichia coli O73		
Escherichia coli O85		
Escherichia coli O99		
Francisella novicida OPS		
Francisella tularensis OPS		
Galactomannan DAVANT (160102)		
Klebsiella O1 OPS	49.68	24.00
Klebsiella O12 OPS		
Klebsiella O2a OPS	29.61	
Klebsiella O2ac OPS	33.38	
Klebsiella O3 OPS		
Klebsiella O4 OPS		
Klebsiella O5 OPS		
Klebsiella O8 OPS	5.94	85.23
Laminarin		
Neisseria meningitidis Group C		
Proteus mirabilis O10 (HJ4320)		
Proteus mirabilis O11 (9B-m)		
Proteus mirabilis O11 (PrK 24/57)		
Proteus mirabilis O13 (PrK 26/57)		
Proteus mirabilis O14a,14b (PrK 29/57)		
Proteus mirabilis O16 (4652)		
Proteus mirabilis O17 (PrK 32/57)		
Proteus mirabilis O18 (PrK 34/57)		
Proteus mirabilis O20 (PrK 38/57)		
Proteus mirabilis O23a, 23b, 23c (CCUG 10701)		
Proteus mirabilis O23a,b,d (PrK 42/57)		
Proteus mirabilis O26 (PrK 49/57)		
Proteus mirabilis O27 (PrK 50/57)		
Proteus mirabilis O28 (PrK 51/57)		
Proteus mirabilis O29a (PrK 52/57)		
Proteus mirabilis O29a, 29b (2002)		
Proteus mirabilis O33 (D52)	52.01	15.69
Proteus mirabilis O3a, 3c (G1)		
Proteus mirabilis O3ab (S1959)		
Proteus mirabilis O40 (10703)		
Proteus mirabilis O41 (PrK 67/57)		
Proteus mirabilis O43 (PrK 69/57)		
Proteus mirabilis O49 (PrK 75/57)	5.43	
Proteus mirabilis O5 (PrK 12/57)		
Proteus mirabilis O50 (TG332)		
Proteus mirabilis O51 (19011)		
Proteus mirabilis O54a, 54b (10704)		
Proteus mirabilis O54ab (OE)		
Proteus mirabilis O57 (TG319)		
Proteus mirabilis O6 (PrK 14/57)		
Proteus mirabilis O74 (10705, OF)		
Proteus mirabilis O75 (10702, OC)		
Proteus mirabilis O77 (3 B-m)		
Proteus mirabilis O8 (TG326)		
Proteus mirabilis O9 (PrK 18/57)		
Proteus myxofaciens O60		
Proteus O56 (genomospecies 4)		
Proteus penneri O17 (16)		
Proteus penneri O31a (26)		
Proteus penneri O31ab (28)		
Proteus penneri O52 (15)		
Proteus penneri O58 (12)		
Proteus penneri O59 (9)		
Proteus penneri O61 (21)		
Proteus penneri O62 (41)		
Proteus penneri O63 (22)		
Proteus penneri O64a, 64b, 64d (39)		
Proteus penneri O64a,b,c (27)		
Proteus penneri O65 (34)		
Proteus penneri O66 (2)		
Proteus penneri O67 (8)		
Proteus penneri O68 (63)		
Proteus penneri O69 (25)		
Proteus penneri O70 (60)		
Proteus penneri O71 (42)		
Proteus penneri O72a, 72b (4)		
Proteus penneri O73a,b (103)		
Proteus penneri O73ac (75)	4.39	
Proteus penneri O8 (106)		

	Gal-3	Gal-3C
Proteus vulgaris O1 (18984)		
Proteus vulgaris O12 (PrK 25/57)		
Proteus vulgaris O13 (8344)		
Proteus vulgaris O15 (PrK 30/57)		
Proteus vulgaris O17 (PrK 33/57)		
Proteus vulgaris O19a (PrK 37/57)		
Proteus vulgaris O2 (OX2)		
Proteus vulgaris O21 (PrK 39/57)		
Proteus vulgaris O22 (PrK 40/57)	8.50	
Proteus vulgaris O24 (PrK 47/57)		
Proteus vulgaris O25 (PrK 48/57)	6.53	
Proteus vulgaris O34 (4669)		
Proteus vulgaris O37a,b (PrK 63/57)		
Proteus vulgaris O37a,c (PrK 72/57)	5.12	
Proteus vulgaris O4 (PrK 9/57)		
Proteus vulgaris O44 (PrK 67/57)		
Proteus vulgaris O45 (4680)	6.95	25.84
Proteus vulgaris O47 (PrK 73/57)	1.84	72.03
Proteus vulgaris O53 (TG 276-10)		
Proteus vulgaris O54a,54c (TG 103)		
Proteus vulgaris O55 (TG 155)		
Proteus vulgaris O65 (TG 251)		
Proteus vulgaris O76 (HSC438)		
Providencia alcalifaciens O19		
Providencia alcalifaciens O19		
Providencia alcalifaciens O19		
Providencia alcalifaciens O21		
Providencia alcalifaciens O23		
Providencia alcalifaciens O27		
Providencia alcalifaciens O29		
Providencia alcalifaciens O30		
Providencia alcalifaciens O32		
Providencia alcalifaciens O36		
Providencia alcalifaciens O39		
Providencia alcalifaciens O5	0.68	4.15
Providencia alcalifaciens O6	2.62	56.15
Providencia rustigianii O14		
Providencia rustigianii O16		
Providencia rustigianii O34		
Providencia stuartii O18		
Providencia stuartii O20		
Providencia stuartii O4		
Providencia stuartii O43		
Providencia stuartii O44		
Providencia stuartii O47		
Providencia stuartii O47, Core 9		
Providencia stuartii O49		
Providencia stuartii O49, Core 1		
Providencia stuartii O52		
Providencia stuartii O57	3.23	
Pseudomonas aerguinosa O1 (Fisher immunotype 4)		
Pseudomonas aerguinosa O10		
Pseudomonas aerguinosa O10 10a,10b		
Pseudomonas aerguinosa O10 10a,10c		
Pseudomonas aerguinosa O11 11a,11b		
Pseudomonas aerguinosa O12 12		
Pseudomonas aerguinosa O13 (Sandvik serotype II)		
Pseudomonas aerguinosa O13 13a,13c		
Pseudomonas aerguinosa O14 14		
Pseudomonas aerguinosa O15 15		
Pseudomonas aerguinosa O2 (2a),2d,2f		
Pseudomonas aerguinosa O2 (Fisher immunotype 3)		
Pseudomonas aerguinosa O2 2a,2b		
Pseudomonas aerguinosa O2 2a,2b,2e	11.76	
Pseudomonas aerguinosa O2 2a,2d		
Pseudomonas aerguinosa O2 Immuno 7	5.23	
Pseudomonas aerguinosa O3 3a,3b		
Pseudomonas aerguinosa O3 3a,3b,3c	3.36	
Pseudomonas aerguinosa O3 3a,3d		
Pseudomonas aerguinosa O4 (Habs serotype 4)		
Pseudomonas aerguinosa O4 4a,4c		
Pseudomonas aerguinosa O6 6a		
Pseudomonas aerguinosa O6 6a,6c		
Pseudomonas aerguinosa O6 Immuno 1		
Pseudomonas aerguinosa O6a (Habs serotype6, fraction IIa)		
Pseudomonas aerguinosa O6a (Habs serotype6, fraction IIb)		
Pseudomonas aerguinosa O7 7a,7b,7c		
Pseudomonas aerguinosa O7 7a,7b,7d		
Pseudomonas aerguinosa O7 7a,7d		
Pseudomonas aerguinosa O9 (9a, 9b, 9d)	7.57	
Salmonella enterica abortus equi LPS		
Salmonella enterica O17		
Salmonella enterica O28		
Salmonella enterica O47		
Salmonella enterica O55		
Salmonella enteritidis dodeca saccharide		
Salmonella enteritidis LPS		
Salmonella typhimurium dodeca saccharide		
Salmonella typhimurium LPS		
Salmonella typhimurium SL 11881 (Re mut)		
Salmonella typhimurium SL 684 (Rc mut)		
Salmonella typhimurium TV 119 (Ra mut)		
Salmonella typhosa LPS		
Serratia marcescens LPS		
Shigella bodyii type10		
Shigella bodyii type2	5.72	
Shigella bodyii type4		
Shigella boydii type 1	4.46	
Shigella boydii type 11	4.48	
Shigella boydii type 12		
Shigella boydii type 13		
Shigella boydii type 14		
Shigella boydii type 15		
Shigella boydii type 16		
Shigella boydii type 17		

	Gal-3	Gal-3C
Shigella boydii type 17		
Shigella boydii type 18		
Shigella boydii type 3		
Shigella boydii type 5		
Shigella boydii type 6		
Shigella boydii type 7		
Shigella boydii type 8		
Shigella boydii type 9		
Shigella dysennteriae type 11		
Shigella dysennteriae type 13		
Shigella dysennteriae type 3		
Shigella dysennteriae type 8		
Shigella dysenteriae type 1		
Shigella dysenteriae type 2		
Shigella dysenteriae type 4		
Shigella dysenteriae type 5		
Shigella dysenteriae type 6 SR-strain		
Shigella dysenteriae type 7		
Shigella dysenteriae type 8 (Russian)		
Shigella dysenteriae type 9		
Shigella flexneri type 1a	11.26	
Shigella flexneri type 1b		
Shigella flexneri type 2a	6.30	
Shigella flexneri type 2b		
Shigella flexneri type 3a		
Shigella flexneri type 3b		
Shigella flexneri type 4a		
Shigella flexneri type 4b		
Shigella flexneri type 5b		
Shigella flexneri type 6		
Shigella flexneri type 6a		
Shigella flexneri type X		
Streptococcus pneumoniae type 1 (Danish type 1)	3.39	
Streptococcus pneumoniae type 12 (Danish type 12F)		
Streptococcus pneumoniae type 14 (Danish type 14)	2.01	100.00
Streptococcus pneumoniae type 17 (Danish type 17F)		
Streptococcus pneumoniae type 19 (Danish type 19F)	8.44	
Streptococcus pneumoniae type 2 (Danish type 2)		
Streptococcus pneumoniae type 20 (Danish type 20)		
Streptococcus pneumoniae type 22 (Danish type 22F)	5.50	
Streptococcus pneumoniae type 23 (Danish type 23F)		
Streptococcus pneumoniae type 26 (Danish type 6B)	19.05	
Streptococcus pneumoniae type 3 (Danish type 3)	3.46	
Streptococcus pneumoniae type 34 (Danish type 10A)		
Streptococcus pneumoniae type 4 (Danish type 4)		
Streptococcus pneumoniae type 43 (Danish type 11A)	0.24	2.02
Streptococcus pneumoniae type 5 (Danish type 5)	9.66	
Streptococcus pneumoniae type 51 (Danish type 7F)		
Streptococcus pneumoniae type 54 (Danish type 15B)	2.57	69.68
Streptococcus pneumoniae type 56 (Danish type 18C)		
Streptococcus pneumoniae type 57 (Danish type 19A)		
Streptococcus pneumoniae type 68 (Danish type 9V)		
Streptococcus pneumoniae type 70 (Danish type 33F)	7.66	52.77
Streptococcus pneumoniae type 8 (Danish type 8)		
Streptococcus pneumoniae type 9 (Danish type 9N)		
Yeast Mannan		
Yeast Mannan Sigma M-3640		
Yersinia pestis 1146-25		
Yersinia pestis 260(11)-37C-186		
Yersinia pestis 260(11)-37C-187		
Yersinia pestis 260(11)-37C-416		
Yersinia pestis 260(11)-37C-417		
Yersinia pestis I-2377-25C		
Yersinia pestis I-2377-37C		
Yersinia pestis KM218-6C		
Yersinia pestis KM260(11)-6C		
Yersinia pestis KM260(11)-wabC/waaL		
Yersinia pestis KM260(11)-wabD/waaL		
Yersinia pestis KM260(11)-yjhW-6C		
Yersinia pestis P-1680-25C		
Yersinia pestis P-1680-37C		
Yersinia pestis, 0KM218-37		
Yersinia pestis, 1146-25		
Yersinia pestis, 1146-37		
Yersinia pestis, 1146-37		
Yersinia pestis, 11M-25		
Yersinia pestis, 11M-37		
Yersinia pestis, KIMD1-25		
Yersinia pestis, KIMD1-37		
Yersinia pestis, KM218-25		
Yersinia pestis, KM218-25		
Yersinia pestis, KM218-37		
Yersinia pestis, KM260(11)-Δ0186		
Yersinia pestis, KM260(11)-Δ0186		
Yersinia pestis, KM260(11)-Δ0187		
Yersinia pestis, KM260(11)-Δ0187		
Yersinia pestis, KM260(11)-ΔpmrF		
Yersinia pestis, KM260(11)-ΔpmrF		
Yersinia pestis, KM260(11)-Δrfe		
Yersinia pestis, KM260(11)-Δrfe		
Yersinia pestis, KM260(11)-ΔwaaL		
Yersinia pestis, KM260(11)-ΔwaaQ		
Yersinia pestis, KM260(11)-ΔwaaQ		
Yersinia pestis, KM260(11)-25		
Yersinia pestis, KM260(11)-25		
Yersinia pestis, KM260(11)-37		
Yersinia pseudotuberculosis 85pCad-20C		
Yersinia pseudotuberculosis 85pCad-37C		
Yersinia pseudotuberculosis O:2a		
Yersinia pseudotuberculosis O:2a-dhmA		
Yersinia pseudotuberculosis O:2c		
Yersinia pseudotuberculosis O:3		
Yersinia pseudotuberculosis O:4b		

Supplementary Figure 2. Heat map of Gal-3 and Gal-3C binding toward glycans presented on the microbial glycan microarray. K_D (red) and unsaturated binding (% max) values (blue) for Gal-3 and Gal-3C are shown. The heat map from darker red (low K_D) to light red (high K_D). For the unsaturated binding, the heat map from light blue (low % max) to darker blue (high % max).
