# Supplementary material for: Full-Length Galectin-3 Is Required for High Affinity Microbial Interactions and Antimicrobial Activity
Source: Front Microbiol. 2021 Oct 8;12:731026. doi: 10.3389/fmicb.2021.731026 (PMC8531552; doi:10.3389/fmicb.2021.731026)
Supplement: Supplementary file 3 [file Data_Sheet_3.PDF]

Wu et al. Supplementary Table 1 (CFG).

Glycan Number	Structure	Gal-3 K_D	Gal-3 % max	Gal-3C K_D	Gal-3C % max
1	Gala-Sp8				
2	Glca-Sp8				
3	Mana-Sp8				
4	GalNAca-Sp8				
5	GalNAca-Sp15				
6	Fuca-Sp8				
7	Fuca-Sp9				
8	Rhaa-Sp8				
9	Neu5Aca-Sp8				
10	Neu5Aca-Sp11				
11	Neu5Acb-Sp8				
12	Galb-Sp8				
13	Glcb-Sp8				
14	Manb-Sp8				
15	GalNAcb-Sp8				
16	GlcNAcb-Sp0				
17	GlcNAcb-Sp8				
18	GlcN(Gc)b-Sp8				
19	Galb1-4GlcNAcb1-6(Galb1-4GlcNAcb1-3)GalNAca-Sp8		11.58		8.2
20	Galb1-4GlcNAcb1-6(Galb1-4GlcNAcb1-3)GalNAc-Sp14		6.46		
21	GlcNAcb1-6(GlcNAcb1-4)(GlcNAcb1-3)GlcNAc-Sp8				
22	6S(3S)Galb1-4(6S)GlcNAcb-Sp0				
23	6S(3S)Galb1-4GlcNAcb-Sp0				
24	(3S)Galb1-4(Fuca1-3)(6S)Glc-Sp0				
25	(3S)Galb1-4Glcb-Sp8	3.8			
26	(3S)Galb1-4(6S)Glcb-Sp0	2.0			11.52
27	(3S)Galb1-4(6S)Glcb-Sp8	2.8			11.47
28	(3S)Galb1-3(Fuca1-4)GlcNAcb-Sp8				
29	(3S)Galb1-3GalNAca-Sp8				
30	(3S)Galb1-3GlcNAcb-Sp0	2.4			21.21
31	(3S)Galb1-3GlcNAcb-Sp8	4.3			20.87
32	(3S)Galb1-4(Fuca1-3)GlcNAc-Sp0				
33	(3S)Galb1-4(Fuca1-3)GlcNAc-Sp8				
34	(3S)Galb1-4(6S)GlcNAcb-Sp0	2.2			21.78
35	(3S)Galb1-4(6S)GlcNAcb-Sp8	3.4			23.81
36	(3S)Galb1-4GlcNAcb-Sp0	3.7			23.38
37	(3S)Galb1-4GlcNAcb-Sp8	1.9			20.22
38	(3S)Galb-Sp8				
39	(6S)(4S)Galb1-4GlcNAcb-Sp0				
40	(4S)Galb1-4GlcNAcb-Sp8				
41	(6P)Mana-Sp8				
42	(6S)Galb1-4Glcb-Sp0				
43	(6S)Galb1-4Glcb-Sp8				
44	(6S)Galb1-4GlcNAcb-Sp8				
45	(6S)Galb1-4(6S)Glcb-Sp8				
46	Neu5Aca2-3(6S)Galb1-4GlcNAcb-Sp8				
47	(6S)GlcNAcb-Sp8				
48	Neu5,9Ac2a-Sp8				
49	Neu5,9Ac2a2-6Galb1-4GlcNAcb-Sp8				
50	Mana1-6(Mana1-3)Manb1-4GlcNAcb1-4GlcNAcb-Sp12				
51	Mana1-6(Mana1-3)Manb1-4GlcNAcb1-4GlcNAcb-Sp13				
52	GlcNAcb1-2Mana1-6(GlcNAcb1-2Mana1-3)Manb1-4GlcNAcb1-4GlcNAcb-Sp12				
53	GlcNAcb1-2Mana1-6(GlcNAcb1-2Mana1-3)Manb1-4GlcNAcb1-4GlcNAcb-Sp13				
54	Galb1-4GlcNAcb1-2Mana1-6(Galb1-4GlcNAcb1-2Mana1-3)Manb1-4GlcNAcb1-4GlcNAcb-Sp12		36.49		10.91
55	Neu5Aca2-6Galb1-4GlcNAcb1-2Mana1-6(Neu5Aca2-6Galb1-4GlcNAcb1-2Mana1-3)Manb1-4GlcNAcb1-4GlcNAcb-Sp12				
56	Neu5Aca2-6Galb1-4GlcNAcb1-2Mana1-6(Neu5Aca2-6Galb1-4GlcNAcb1-2Man-a1-3)Manb1-4GlcNAcb1-4GlcNAcb-Sp21				
57	Neu5Aca2-6Galb1-4GlcNAcb1-2Mana1-6(Neu5Aca2-6Galb1-4GlcNAcb1-2Mana1-3)Manb1-4GlcNAcb1-4GlcNAcb-Sp24				
58	Fuca1-2Galb1-3GalNAcb1-3Gala-Sp9				

**59**	Fuca1-2Galb1-3GalNAcb1-3Gala1-4Galb1-4Glcb-Sp9				
**60**	Fuca1-2Galb1-3(Fuca1-4)GlcNAcb-Sp8				
**61**	Fuca1-2Galb1-3GalNAca-Sp8				
**62**	Fuca1-2Galb1-3GalNAca-Sp14				
**63**	Fuca1-2Galb1-3GalNAcb1-4(Neu5Aca2-3)Galb1-4Glcb-Sp0				
**64**	Fuca1-2Galb1-3GalNAcb1-4(Neu5Aca2-3)Galb1-4Glcb-Sp9				
**65**	Fuca1-2Galb1-3GlcNAcb1-3Galb1-4Glcb-Sp8		37.41		18.41
**66**	Fuca1-2Galb1-3GlcNAcb1-3Galb1-4Glcb-Sp10	7.4			25.6
**67**	Fuca1-2Galb1-3GlcNAcb-Sp0		19.05		
**68**	Fuca1-2Galb1-3GlcNAcb-Sp8		13.37		
**69**	Fuca1-2Galb1-4(Fuca1-3)GlcNAcb1-3Galb1-4(Fuca1-3)GlcNAcb-Sp0				
**70**	Fuca1-2Galb1-4(Fuca1-3)GlcNAcb1-3Galb1-4(Fuca1-3)GlcNAcb1-3Galb1-4(Fuca1-3)GlcNAcb-Sp0				
**71**	Fuca1-2Galb1-4(Fuca1-3)GlcNAcb-Sp0				
**72**	Fuca1-2Galb1-4(Fuca1-3)GlcNAcb-Sp8				
**73**	Fuca1-2Galb1-4GlcNAcb1-3Galb1-4GlcNAcb-Sp0		58.5		64.01
**74**	Fuca1-2Galb1-4GlcNAcb1-3Galb1-4GlcNAcb1-3Galb1-4GlcNAcb-Sp0	1.9		8.1	
**75**	Fuca1-2Galb1-4GlcNAcb-Sp0		15.3		
**76**	Fuca1-2Galb1-4GlcNAcb-Sp8		16.6		
**77**	Fuca1-2Galb1-4Glcb-Sp0		8.88		
**78**	Fuca1-2Galb-Sp8				
**79**	Fuca1-3GlcNAcb-Sp8				
**80**	Fuca1-4GlcNAcb-Sp8				
**81**	Fucb1-3GlcNAcb-Sp8				
**82**	GalNAca1-3(Fuca1-2)Galb1-3GlcNAcb-Sp0	6.7			26.05
**83**	GalNAca1-3(Fuca1-2)Galb1-4(Fuca1-3)GlcNAcb-Sp0				
**84**	(3S)Galb1-4(Fuca1-3)Glcb-Sp0				
**85**	GalNAca1-3(Fuca1-2)Galb1-4GlcNAcb-Sp0	1.7			46.16
**86**	GalNAca1-3(Fuca1-2)Galb1-4GlcNAcb-Sp8	2.2			52.53
**87**	GalNAca1-3(Fuca1-2)Galb1-4Glcb-Sp0		38.53		23.22
**88**	GlcNAcb1-3Galb1-3GalNAca-Sp8				
**89**	GalNAca1-3(Fuca1-2)Galb-Sp8				
**90**	GalNAca1-3(Fuca1-2)Galb-Sp18				
**91**	GalNAca1-3GalNAcb-Sp8				
**92**	GalNAca1-3Galb-Sp8				
**93**	GalNAca1-4(Fuca1-2)Galb1-4GlcNAcb-Sp8		27.62		10.97
**94**	GalNAcb1-3GalNAca-Sp8				
**95**	GalNAcb1-3(Fuca1-2)Galb-Sp8				
**96**	GalNAcb1-3Gala1-4Galb1-4GlcNAcb-Sp0				
**97**	GalNAcb1-4(Fuca1-3)GlcNAcb-Sp0				
**98**	GalNAcb1-4GlcNAcb-Sp0				
**99**	GalNAcb1-4GlcNAcb-Sp8				
**100**	Gala1-2Galb-Sp8				
**101**	Gala1-3(Fuca1-2)Galb1-3GlcNAcb-Sp0	2.1			66.97
**102**	Gala1-3(Fuca1-2)Galb1-3GlcNAcb-Sp8	1.5			57.19
**103**	Gala1-3(Fuca1-2)Galb1-4(Fuca1-3)GlcNAcb-Sp0				
**104**	Gala1-3(Fuca1-2)Galb1-4(Fuca1-3)GlcNAcb-Sp8				
**105**	Gala1-3(Fuca1-2)Galb1-4GlcNAc-Sp0	0.9		5.2	
**106**	Gala1-3(Fuca1-2)Galb1-4Glcb-Sp0	2.4			54.4
**107**	Gala1-3(Fuca1-2)Galb-Sp8				
**108**	Gala1-3(Fuca1-2)Galb-Sp18				
**109**	Gala1-4(Gala1-3)Galb1-4GlcNAcb-Sp8				
**110**	Gala1-3GalNAca-Sp8				
**111**	Gala1-3GalNAca-Sp16				
**112**	Gala1-3GalNAcb-Sp8				
**113**	Gala1-3Galb1-4(Fuca1-3)GlcNAcb-Sp8		25.08		10.86
**114**	Gala1-3Galb1-3GlcNAcb-Sp0		73.38		30.59
**115**	Gala1-3Galb1-4GlcNAcb-Sp8		13.7		
**116**	Gala1-3Galb1-4Glcb-Sp0		22.4		8.83
**117**	Gala1-3Galb1-4Glc-Sp10				
**118**	Gala1-3Galb-Sp8				
**119**	Gala1-4(Fuca1-2)Galb1-4GlcNAcb-Sp8				
**120**	Gala1-4Galb1-4GlcNAcb-Sp0				
**121**	Gala1-4Galb1-4GlcNAcb-Sp8				
**122**	Gala1-4Galb1-4Glcb-Sp0				

**123**	Gala1-4GlcNAcb-Sp8				
**124**	Gala1-6Glcb-Sp8				
**125**	Galb1-2Galb-Sp8				
**126**	Galb1-3(Fuca1-4)GlcNAcb1-3Galb1-4(Fuca1-3)GlcNAcb-Sp0				
**127**	Galb1-3GlcNAcb1-3Galb1-4(Fuca1-3)GlcNAcb-Sp0		7.69		
**128**	Galb1-3(Fuca1-4)GlcNAc-Sp0				
**129**	Galb1-3(Fuca1-4)GlcNAc-Sp8				
**130**	Fuca1-4(Galb1-3)GlcNAcb-Sp8				
**131**	Galb1-4GlcNAcb1-6GalNAca-Sp8				
**132**	Galb1-4GlcNAcb1-6GalNAc-Sp14				
**133**	GlcNAcb1-6(Galb1-3)GalNAca-Sp8				
**134**	Neu5Aca2-6(Galb1-3)GalNAca-Sp8				
**135**	Neu5Aca2-6(Galb1-3)GalNAca-Sp14				
**136**	Neu5Acb2-6(Galb1-3)GalNAca-Sp8				
**137**	Neu5Aca2-6(Galb1-3)GlcNAcb1-4Galb1-4Glcb-Sp10				
**138**	Galb1-3GalNAca-Sp8				
**139**	Galb1-3GalNAca-Sp14				
**140**	Galb1-3GalNAca-Sp16				
**141**	Galb1-3GalNAcb-Sp8				
**142**	Galb1-3GalNAcb1-3Gala1-4Galb1-4Glcb-Sp0				
**143**	Galb1-3GalNAcb1-4(Neu5Aca2-3)Galb1-4Glcb-Sp0				
**144**	Galb1-3GalNAcb1-4Galb1-4Glcb-Sp8				
**145**	Galb1-3Galb-Sp8				
**146**	Galb1-3GlcNAcb1-3Galb1-4GlcNAcb-Sp0		36.11		21.53
**147**	Galb1-3GlcNAcb1-3Galb1-4Glcb-Sp10	7.7			15.0
**148**	Galb1-3GlcNAcb-Sp0		10.72		
**149**	Galb1-3GlcNAcb-Sp8				
**150**	Galb1-4(Fuca1-3)GlcNAcb-Sp0				
**151**	Galb1-4(Fuca1-3)GlcNAcb-Sp8				
**152**	Galb1-4(Fuca1-3)GlcNAcb1-3Galb1-4(Fuca1-3)GlcNAcb-Sp0				
**153**	Galb1-4(Fuca1-3)GlcNAcb1-3Galb1-4(Fuca1-3)GlcNAcb1-3Galb1-4(Fuca1-3)GlcNAcb-Sp0				
**154**	Galb1-4(6S)Glcb-Sp0				
**155**	Galb1-4(6S)Glcb-Sp8				
**156**	Galb1-4GalNAca1-3(Fuca1-2)Galb1-4GlcNAcb-Sp8	2.1			74.07
**157**	Galb1-4GalNAcb1-3(Fuca1-2)Galb1-4GlcNAcb-Sp8				
**158**	Galb1-4GlcNAcb1-3GalNAca-Sp8				
**159**	Galb1-4GlcNAcb1-3GalNAc-Sp14		6.58		
**160**	Galb1-4GlcNAcb1-3Galb1-4(Fuca1-3)GlcNAcb1-3Galb1-4(Fuca1-3)GlcNAcb-Sp0				
**161**	Galb1-4GlcNAcb1-3Galb1-4GlcNAcb1-3Galb1-4GlcNAcb-Sp0	6.2			56.02
**162**	Galb1-4GlcNAcb1-3Galb1-4GlcNAcb-Sp0	7.0			70.49
**163**	Galb1-4GlcNAcb1-3Galb1-4Glcb-Sp0	7.3			29.74
**164**	Galb1-4GlcNAcb1-3Galb1-4Glcb-Sp8		50.81		28.64
**165**	Galb1-4GlcNAcb1-6(Galb1-3)GalNAca-Sp8				
**166**	Galb1-4GlcNAcb1-6(Galb1-3)GalNAc-Sp14				
**167**	Galb1-4GlcNAcb-Sp0				
**168**	Galb1-4GlcNAcb-Sp8				
**169**	Galb1-4GlcNAcb-Sp23				
**170**	Galb1-4Glcb-Sp0				
**171**	Galb1-4Glcb-Sp8				
**172**	GlcNAca1-3Galb1-4GlcNAcb-Sp8		7.91		
**173**	GlcNAca1-6Galb1-4GlcNAcb-Sp8				
**174**	GlcNAcb1-2Galb1-3GalNAca-Sp8				
**175**	GlcNAcb1-6(GlcNAcb1-3)GalNAca-Sp8				
**176**	GlcNAcb1-6(GlcNAcb1-3)GalNAca-Sp14				
**177**	GlcNAcb1-6(GlcNAcb1-3)Galb1-4GlcNAcb-Sp8				
**178**	GlcNAcb1-3GalNAca-Sp8				
**179**	GlcNAcb1-3GalNAca-Sp14				
**180**	GlcNAcb1-3Galb-Sp8				
**181**	GlcNAcb1-3Galb1-4GlcNAcb-Sp0		16.61		11.59
**182**	GlcNAcb1-3Galb1-4GlcNAcb-Sp8		35.22		22.14
**183**	GlcNAcb1-3Galb1-4GlcNAcb1-3Galb1-4GlcNAcb-Sp0	3.8			73.87
**184**	GlcNAcb1-3Galb1-4Glcb-Sp0		13.56		
**185**	GlcNAcb1-4-MDPLys				

**186**	GlcNAcb1-6(GlcNAcb1-4)GalNAca-Sp8				
**187**	GlcNAcb1-4Galb1-4GlcNAcb-Sp8				
**188**	GlcNAcb1-4GlcNAcb1-4GlcNAcb1-4GlcNAcb1-4GlcNAcb1-4GlcNAcb1-Sp8				
**189**	GlcNAcb1-4GlcNAcb1-4GlcNAcb1-4GlcNAcb1-4GlcNAcb1-Sp8				
**190**	GlcNAcb1-4GlcNAcb1-4GlcNAcb-Sp8				
**191**	GlcNAcb1-6GalNAca-Sp8				
**192**	GlcNAcb1-6GalNAca-Sp14				
**193**	GlcNAcb1-6Galb1-4GlcNAcb-Sp8				
**194**	Glca1-4Glcb-Sp8				
**195**	Glca1-4Glca-Sp8				
**196**	Glca1-6Glca1-6Glcb-Sp8				
**197**	Glcb1-4Glcb-Sp8				
**198**	Glcb1-6Glcb-Sp8				
**199**	G-ol-Sp8				
**200**	GlcAa-Sp8				
**201**	GlcAb-Sp8				
**202**	GlcAb1-3Galb-Sp8				
**203**	GlcAb1-6Galb-Sp8				
**204**	KDNa2-3Galb1-3GlcNAcb-Sp0		14.06		
**205**	KDNa2-3Galb1-4GlcNAcb-Sp0		7.36		
**206**	Mana1-2Mana1-2Mana1-3Mana-Sp9				
**207**	Mana1-2Mana1-6(Mana1-2Mana1-3)Mana-Sp9				
**208**	Mana1-2Mana1-3Mana-Sp9				
**209**	Mana1-6(Mana1-3)Mana-Sp9				
**210**	Mana1-2Mana1-2Mana1-6(Mana1-3)Mana-Sp9				
**211**	Mana1-6(Mana1-3)Mana1-6(Mana1-2Mana1-3)Manb1-4GlcNAcb1-4GlcNAcb-Sp12				
**212**	Mana1-6(Mana1-3)Mana1-6(Mana1-3)Manb1-4GlcNAcb1-4GlcNAcb-Sp12				
**213**	Manb1-4GlcNAcb-Sp0				
**214**	Neu5Aca2-3Galb1-4GlcNAcb1-3Galb1-4(Fuca1-3)GlcNAcb-Sp0				
**215**	(3S)Galb1-4(Fuca1-3)(6S)GlcNAcb-Sp8				
**216**	Fuca1-2(6S)Galb1-4GlcNAcb-Sp0				
**217**	Fuca1-2Galb1-4(6S)GlcNAcb-Sp8		16.71		7.29
**218**	Fuca1-2(6S)Galb1-4(6S)Glcb-Sp0				
**219**	Neu5Aca2-3Galb1-3GalNAca-Sp8				
**220**	Neu5Aca2-3Galb1-3GalNAca-Sp14				
**221**	GalNAcb1-4(Neu5Aca2-8Neu5Aca2-8Neu5Aca2-8Neu5Aca2-3)Galb1-4Glcb-Sp0				
**222**	GalNAcb1-4(Neu5Aca2-8Neu5Aca2-8Neu5Aca2-3)Galb1-4Glcb-Sp0				
**223**	Neu5Aca2-8Neu5Aca2-8Neu5Aca2-3Galb1-4Glcb-Sp0				
**224**	GalNAcb1-4(Neu5Aca2-8Neu5Aca2-3)Galb1-4Glcb-Sp0				
**225**	Neu5Aca2-8Neu5Aca2-8Neu5Aca-Sp8				
**226**	GalNAcb1-4(Neu5Aca2-3)Galb1-4GlcNAcb-Sp0				
**227**	GalNAcb1-4(Neu5Aca2-3)Galb1-4GlcNAcb-Sp8				
**228**	GalNAcb1-4(Neu5Aca2-3)Galb1-4Glcb-Sp0				
**229**	Neu5Aca2-3Galb1-3GalNAcb1-4(Neu5Aca2-3)Galb1-4Glcb-Sp0				
**230**	Neu5Aca2-6(Neu5Aca2-3)GalNAca-Sp8				
**231**	Neu5Aca2-3GalNAca-Sp8				
**232**	Neu5Aca2-3GalNAcb1-4GlcNAcb-Sp0		8.04		
**233**	Neu5Aca2-3Galb1-3(6S)GlcNAc-Sp8				
**234**	Neu5Aca2-3Galb1-3(Fuca1-4)GlcNAcb-Sp8				
**235**	Neu5Aca2-3Galb1-3(Fuca1-4)GlcNAcb1-3Galb1-4(Fuca1-3)GlcNAcb-Sp0				
**236**	Neu5Aca2-3Galb1-4(Neu5Aca2-3Galb1-3)GlcNAcb-Sp8				
**237**	Neu5Aca2-3Galb1-3(6S)GalNAca-Sp8				
**238**	Neu5Aca2-6(Neu5Aca2-3Galb1-3)GalNAca-Sp8				
**239**	Neu5Aca2-6(Neu5Aca2-3Galb1-3)GalNAca-Sp14				
**240**	Neu5Aca2-3Galb-Sp8				
**241**	Neu5Aca2-3Galb1-3GalNAcb1-3Gala1-4Galb1-4Glcb-Sp0				
**242**	Neu5Aca2-3Galb1-3GlcNAcb1-3Galb1-4GlcNAcb-Sp0	5.8			28.73
**243**	Fuca1-2(6S)Galb1-4Glcb-Sp0				
**244**	Neu5Aca2-3Galb1-3GlcNAcb-Sp0	8.0			
**245**	Neu5Aca2-3Galb1-4(6S)GlcNAcb-Sp8	7.1			15.88

**246**	Neu5Aca2-3Galb1-4(Fuca1-3)(6S)GlcNAcb-Sp8				
**247**	Neu5Aca2-3Galb1-4(Fuca1-3)GlcNAcb1-3Galb1-4(Fuca1-3)GlcNAcb1-3Galb1-4(Fuca1-3)GlcNAcb-Sp0				
**248**	Neu5Aca2-3Galb1-4(Fuca1-3)GlcNAcb-Sp0				
**249**	Neu5Aca2-3Galb1-4(Fuca1-3)GlcNAcb-Sp8				
**250**	Neu5Aca2-3Galb1-4(Fuca1-3)GlcNAcb1-3Galb-Sp8				
**251**	Neu5Aca2-3Galb1-4(Fuca1-3)GlcNAcb1-3Galb1-4GlcNAcb-Sp8				
**252**	Neu5Aca2-3Galb1-4GlcNAcb1-3Galb1-4GlcNAcb1-3Galb1-4GlcNAcb-Sp0				
**253**	Neu5Aca2-3Galb1-4GlcNAcb-Sp0		6.73		
**254**	Neu5Aca2-3Galb1-4GlcNAcb-Sp8		9.72		
**255**	Neu5Aca2-3Galb1-4GlcNAcb1-3Galb1-4GlcNAcb-Sp0		20.85		12.18
**256**	Fuca1-2Galb1-4(6S)Glcb-Sp0		12.42		
**257**	Neu5Aca2-3Galb1-4Glcb-Sp0				
**258**	Neu5Aca2-3Galb1-4Glcb-Sp8				
**259**	Neu5Aca2-6GalNAca-Sp8				
**260**	Neu5Aca2-6GalNAcb1-4GlcNAcb-Sp0				
**261**	Neu5Aca2-6Galb1-4(6S)GlcNAcb-Sp8				
**262**	Neu5Aca2-6Galb1-4GlcNAcb-Sp8				
**263**	Neu5Aca2-6Galb1-4GlcNAcb1-3Galb1-4(Fuca1-3)GlcNAcb1-3Galb1-4(Fuca1-3)GlcNAcb-Sp0				
**264**	Neu5Aca2-6Galb1-4GlcNAcb1-3Galb1-4GlcNAcb-Sp0		25.46		27.25
**265**	Neu5Aca2-6Galb1-4Glcb-Sp0				
**266**	Neu5Aca2-6Galb1-4Glcb-Sp8				
**267**	Neu5Aca2-6Galb-Sp8				
**268**	Neu5Aca2-8Neu5Aca-Sp8				
**269**	Neu5Aca2-8Neu5Aca2-3Galb1-4Glcb-Sp0				
**270**	Galb1-3(Fuca1-4)GlcNAcb1-3Galb1-3(Fuca1-4)GlcNAcb-Sp0				
**271**	Neu5Acb2-6GalNAca-Sp8				
**272**	Neu5Acb2-6Galb1-4GlcNAcb-Sp8				
**273**	Neu5Gca2-3Galb1-3(Fuca1-4)GlcNAcb-Sp0				
**274**	Neu5Gca2-3Galb1-3GlcNAcb-Sp0		18.8		
**275**	Neu5Gca2-3Galb1-4(Fuca1-3)GlcNAcb-Sp0				
**276**	Neu5Gca2-3Galb1-4GlcNAcb-Sp0		11.19		
**277**	Neu5Gca2-3Galb1-4Glcb-Sp0				
**278**	Neu5Gca2-6GalNAca-Sp0				
**279**	Neu5Gca2-6Galb1-4GlcNAcb-Sp0				
**280**	Neu5Gca-Sp8				
**281**	Neu5Aca2-3Galb1-4GlcNAcb1-6(Galb1-3)GalNAca-Sp14				
**282**	Galb1-3GlcNAcb1-3Galb1-3GlcNAcb-Sp0		45.82		24.65
**283**	Galb1-4(Fuca1-3)(6S)GlcNAcb-Sp0				
**284**	Galb1-4(Fuca1-3)(6S)Glcb-Sp0				
**285**	Galb1-4(Fuca1-3)GlcNAcb1-3Galb1-3(Fuca1-4)GlcNAcb-Sp0				
**286**	Galb1-4GlcNAcb1-3Galb1-3GlcNAcb-Sp0		30.74		20.68
**287**	Neu5Aca2-3Galb1-3GlcNAcb1-3Galb1-3GlcNAcb-Sp0	5.9			28.57
**288**	Neu5Aca2-3Galb1-4GlcNAcb1-3Galb1-3GlcNAcb-Sp0		26.03		11.73
**289**	4S(3S)Galb1-4GlcNAcb-Sp0				
**290**	(6S)Galb1-4(6S)GlcNAcb-Sp0				
**291**	(6P)Glcb-Sp10				
**292**	Galb1-3Galb1-4GlcNAcb-Sp8				
**293**	Neu5Aca2-6Galb1-4GlcNAcb1-2Mana1-6(Galb1-4GlcNAcb1-2Mana1-3)Manb1-4GlcNAcb1-4GlcNAcb-Sp12				
**294**	Galb1-4GlcNAcb1-6(Galb1-4GlcNAcb1-3)Galb1-4GlcNAc-Sp0		17.5		16.51
**295**	GlcNAcb1-6(Galb1-4GlcNAcb1-3)Galb1-4GlcNAc-Sp0		9.19		
**296**	Galb1-4GlcNAca1-6Galb1-4GlcNAcb-Sp0		11.12		
**297**	Galb1-4GlcNAcb1-6Galb1-4GlcNAcb-Sp0				
**298**	GalNAcb1-3Galb-Sp8				
**299**	GlcAb1-3GlcNAcb-Sp8				
**300**	Neu5Aca2-6Galb1-4GlcNAcb1-2Mana1-6(GlcNAcb1-2Mana1-3)Manb1-4GlcNAcb1-4GlcNAcb-Sp12				
**301**	GlcNAcb1-3Man-Sp10				
**302**	GlcNAcb1-4GlcNAcb-Sp10				
**303**	GlcNAcb1-4GlcNAcb-Sp12				
**304**	MurNAcb1-4GlcNAcb-Sp10				
**305**	Mana1-6Manb-Sp10				

**306**	Mana1-6(Mana1-3)Mana1-6(Mana1-3)Manb-Sp10				
**307**	Mana1-2Mana1-6(Mana1-3)Mana1-6(Mana1-2Mana1-2Mana1-3)Mana-Sp9				
**308**	Mana1-2Mana1-6(Mana1-2Mana1-3)Mana1-6(Mana1-2Mana1-2Mana1-3)Mana-Sp9				
**309**	Neu5Aca2-3Galb1-4GlcNAcb1-6(Neu5Aca2-3Galb1-3)GalNAca-Sp14		7.11		
**310**	Neu5Aca2-6Galb1-4GlcNAcb1-2Mana1-6(Neu5Aca2-3Galb1-4GlcNAcb1-2Mana1-3)Manb1-4GlcNAcb1-4GlcNAcb-Sp12		14.04		
**311**	Galb1-4GlcNAcb1-2Mana1-6(Neu5Aca2-6Galb1-4GlcNAcb1-2Mana1-3)Manb1-4GlcNAcb1-4GlcNAcb-Sp12				
**312**	Neu5Aca2-8Neu5Aca2-8Neu5Acb-Sp8				
**313**	Neu5Gcb2-6Galb1-4GlcNAc-Sp8				
**314**	Galb1-3GlcNAcb1-2Mana1-6(Galb1-3GlcNAcb1-2Mana1-3)Manb1-4GlcNAcb1-4GlcNAcb-Sp19		6.33		
**315**	Neu5Aca2-3Galb1-4GlcNAcb1-2Mana1-6(Neu5Aca2-3Galb1-4GlcNAcb1-2Mana1-3)Manb1-4GlcNAcb1-4GlcNAcb-Sp12	8.4			24.81
**316**	Neu5Aca2-3Galb1-4GlcNAcb1-2Mana1-6(Neu5Aca2-6Galb1-4GlcNAcb1-2Mana1-3)Manb1-4GlcNAcb1-4GlcNAcb-Sp12		13.56		
**317**	Galb1-4(Fuca1-3)GlcNAcb1-2Mana1-6(Galb1-4(Fuca1-3)GlcNAcb1-2Mana1-3)Manb1-4GlcNAcb1-4GlcNAcb-Sp20				
**318**	Neu5,9Ac2a2-3Galb1-3GlcNAcb-Sp0		8.94		
**319**	Neu5Aca2-6Galb1-4GlcNAcb1-3Galb1-3GlcNAcb-Sp0	4.6			40.47
**320**	Neu5Aca2-3Galb1-3(Fuca1-4)GlcNAcb1-3Galb1-3(Fuca1-4)GlcNAcb-Sp0				
**321**	Neu5Aca2-6Galb1-4GlcNAcb1-3Galb1-4GlcNAcb1-3Galb1-4GlcNAcb-Sp0	2.5			68.39
**322**	Gala1-4Galb1-4GlcNAcb1-3Galb1-4Glcb-Sp0		44.7		29.48
**323**	GalNAcb1-3Gala1-4Galb1-4GlcNAcb1-3Galb1-4Glcb-Sp0		49.08		39.11
**324**	GalNAca1-3(Fuca1-2)Galb1-4GlcNAcb1-3Galb1-4GlcNAcb-Sp0	1.7		8.0	
**325**	GalNAca1-3(Fuca1-2)Galb1-4GlcNAcb1-3Galb1-4GlcNAcb1-3Galb1-4GlcNAcb-Sp0	2.1			62.5
**326**	Neu5Aca2-3Galb1-4(Fuca1-3)GlcNAcb1-6(Neu5Aca2-3Galb1-3)GalNAc-Sp14				
**327**	GlcNAca1-4Galb1-4GlcNAcb1-3Galb1-4GlcNAcb1-3Galb1-4GlcNAcb-Sp0	5.1			70.87
**328**	GlcNAca1-4Galb1-4GlcNAcb-Sp0				
**329**	GlcNAca1-4Galb1-3GlcNAcb-Sp0				
**330**	GlcNAca1-4Galb1-4GlcNAcb1-3Galb1-4Glcb-Sp0		31.09		16.01
**331**	GlcNAca1-4Galb1-4GlcNAcb1-3Galb1-4(Fuca1-3)GlcNAcb1-3Galb1-4(Fuca1-3)GlcNAcb-Sp0				
**332**	GlcNAca1-4Galb1-4GlcNAcb1-3Galb1-4GlcNAcb-Sp0	8.0			41.06
**333**	GlcNAca1-4Galb1-3GalNAc-Sp14				
**334**	Neu5Aca2-6Galb1-4GlcNAcb1-2Mana1-6(Mana1-3)Manb1-4GlcNAcb1-4GlcNAc-Sp12				
**335**	Mana1-6(Neu5Aca2-6Galb1-4GlcNAcb1-2Mana1-3)Manb1-4GlcNAcb1-4GlcNAc-Sp12				
**336**	Neu5Aca2-6Galb1-4GlcNAcb1-2Mana1-6Manb1-4GlcNAcb1-4GlcNAc-Sp12				
**337**	Neu5Aca2-6Galb1-4GlcNAcb1-2Mana1-3Manb1-4GlcNAcb1-4GlcNAc-Sp12				
**338**	Galb1-4GlcNAcb1-2Mana1-3Manb1-4GlcNAcb1-4GlcNAc-Sp12		8.73		
**339**	Galb1-4GlcNAcb1-2Mana1-6Manb1-4GlcNAcb1-4GlcNAc-Sp12		8.53		
**340**	Mana1-6(Galb1-4GlcNAcb1-2Mana1-3)Manb1-4GlcNAcb1-4GlcNAcb-Sp12		7.0		
**341**	GlcNAcb1-2Mana1-6(GlcNAcb1-2Mana1-3)Manb1-4GlcNAcb1-4(Fuca1-6)GlcNAcb-Sp22				
**342**	Galb1-4GlcNAcb1-2Mana1-6(Galb1-4GlcNAcb1-2Mana1-3)Manb1-4GlcNAcb1-4(Fuca1-6)GlcNAcb-Sp22		12.67		
**343**	Galb1-3GlcNAcb1-2Mana1-6(Galb1-3GlcNAcb1-2Mana1-3)Manb1-4GlcNAcb1-4(Fuca1-6)GlcNAcb-Sp22				
**344**	(6S)GlcNAcb1-3Galb1-4GlcNAcb-Sp0				
**345**	KDNa2-3Galb1-4(Fuca1-3)GlcNAc-Sp0				
**346**	KDNa2-6Galb1-4GlcNAc-Sp0				
**347**	KDNa2-3Galb1-4Glc-Sp0				
**348**	KDNa2-3Galb1-3GalNAca-Sp14				
**349**	Fuca1-2Galb1-3GlcNAcb1-2Mana1-6(Fuca1-2Galb1-3GlcNAcb1-2Mana1-3)Manb1-4GlcNAcb1-4GlcNAcb-Sp20	6.51			16.41

**350**	Fuca1-2Galb1-4GlcNAcb1-2Mana1-6(Fuca1-2Galb1-4GlcNAcb1-2Mana1-3)Manb1-4GlcNAcb1-4GlcNAcb-Sp20		36.84		18.95
**351**	Fuca1-2Galb1-4(Fuca1-3)GlcNAcb1-2Mana1-6(Fuca1-2Galb1-4(Fuca1-3)GlcNAcb1-2Mana1-3)Manb1-4GlcNAcb1-4GlcNAb-Sp20				
**352**	Gala1-3Galb1-4GlcNAcb1-2Mana1-6(Gala1-3Galb1-4GlcNAcb1-2Mana1-3)Manb1-4GlcNAcb1-4GlcNAcb-Sp20	2.2			100.0
**353**	Galb1-4GlcNAcb1-2Mana1-6(Mana1-3)Manb1-4GlcNAcb1-4GlcNAcb-Sp12				
**354**	Fuca1-4(Galb1-3)GlcNAcb1-2Mana1-6(Fuca1-4(Galb1-3)GlcNAcb1-2Mana1-3)Manb1-4GlcNAcb1-4(Fuca1-6)GlcNAcb-Sp22				
**355**	Neu5Aca2-6GlcNAcb1-4GlcNAc-Sp21				
**356**	Neu5Aca2-6GlcNAcb1-4GlcNAcb1-4GlcNAc-Sp21				
**357**	Galb1-4(Fuca1-3)GlcNAcb1-6(Fuca1-2Galb1-4GlcNAcb1-3)Galb1-4Glc-Sp21	10.0			
**358**	Galb1-4GlcNAcb1-2Mana1-6(Galb1-4GlcNAcb1-4(Galb1-4GlcNAcb1-2)Mana1-3)Manb1-4GlcNAcb1-4GlcNAc-Sp21		60.32		53.91
**359**	GalNAca1-3(Fuca1-2)Galb1-4GlcNAcb1-2Mana1-6(GalNAca1-3(Fuca1-2)Galb1-4GlcNAcb1-2Mana1-3)Manb1-4GlcNAcb1-4GlcNAcb-Sp20	1.0		4.7	
**360**	Gala1-3(Fuca1-2)Galb1-4GlcNAcb1-2Mana1-6(Gala1-3(Fuca1-2)Galb1-4GlcNAcb1-2Mana1-3)Manb1-4GlcNAcb1-4GlcNAcb-Sp20	0.5		1.7	
**361**	Gala1-3Galb1-4(Fuca1-3)GlcNAcb1-2Mana1-6(Gala1-3Galb1-4(Fuca1-3)GlcNAcb1-2Mana1-3)Manb1-4GlcNAcb1-4GlcNAcb-Sp20				
**362**	GalNAca1-3(Fuca1-2)Galb1-3GlcNAcb1-2Mana1-6(GalNAca1-3(Fuca1-2)Galb1-3GlcNAcb1-2Mana1-3)Manb1-4GlcNAcb1-4GlcNAcb-Sp20	2.8			81.66
**363**	Gala1-3(Fuca1-2)Galb1-3GlcNAcb1-2Mana1-6(Galca1-3(Fuca1-2)Galb1-3GlcNAcb1-2Mana1-3)Manb1-4GlcNAcb1-4GlcNAcb-Sp20	1.7		4.9	
**364**	Fuca1-4(Fuca1-2Galb1-3)GlcNAcb1-2Mana1-3(Fuca1-4(Fuca1-2Galb1-3)GlcNAcb1-2Mana1-3)Manb1-4GlcNAcb1-4GlcNAcb-Sp19				
**365**	Neu5Aca2-3Galb1-4GlcNAcb1-3GalNAc-Sp14		7.28		
**366**	Neu5Aca2-6Galb1-4GlcNAcb1-3GalNAc-Sp14				
**367**	Neu5Aca2-3Galb1-4(Fuca1-3)GlcNAcb1-3GalNAca-Sp14				
**368**	GalNAcb1-4GlcNAcb1-2Mana1-6(GalNAcb1-4GlcNAcb1-2Mana1-3)Manb1-4GlcNAcb1-4GlcNAc-Sp12				
**369**	Galb1-3GalNAca1-3(Fuca1-2)Galb1-4Glc-Sp0		33.43		17.77
**370**	Galb1-3GalNAca1-3(Fuca1-2)Galb1-4GlcNAc-Sp0	2.2			50.75
**371**	Galb1-3GlcNAcb1-3Galb1-4GlcNAcb1-6(Galb1-3GlcNAcb1-3)Galb1-4Glcb-Sp0		56.19		37.99
**372**	Galb1-4(Fuca1-3)GlcNAcb1-6(Galb1-3GlcNAcb1-3)Galb1-4Glc-Sp21				
**373**	Galb1-4GlcNAcb1-6(Fuca1-4(Fuca1-2Galb1-3)GlcNAcb1-3)Galb1-4Glc-Sp21				
**374**	Galb1-4(Fuca1-3)GlcNAcb1-6(Fuca1-4(Fuca1-2Galb1-3)GlcNAcb1-3)Galb1-4Glc-Sp21				
**375**	Galb1-3GlcNAcb1-3Galb1-4(Fuca1-3)GlcNAcb1-6(Galb1-3GlcNAcb1-3)Galb1-4Glc-Sp21				
**376**	Galb1-4GlcNAcb1-6(Galb1-4GlcNAcb1-2)Mana1-6(Galb1-4GlcNAcb1-4(Galb1-4GlcNAcb1-2)Mana1-3)Manb1-4GlcNAcb1-4GlcNAcb-Sp21		84.38		72.19
**377**	GlcNAcb1-2Mana1-6(GlcNAcb1-4(GlcNAcb1-2)Mana1-3)Manb1-4GlcNAcb1-4GlcNAc-Sp21				
**378**	Fuca1-2Galb1-3GalNAca1-3(Fuca1-2)Galb1-4Glcb-Sp0				
**379**	Fuca1-2Galb1-3GalNAca1-3(Fuca1-2)Galb1-4GlcNAcb-Sp0		17.89		12.54
**380**	Galb1-3GlcNAcb1-3GalNAca-Sp14	6.3			
**381**	GalNAca1-3(Fuca1-2)Galb1-3GalNAca1-3(Fuca1-2)Galb1-4GlcNAcb-Sp0	9.2			10.93
**382**	Gala1-3Galb1-3GlcNAcb1-2Mana1-6(Gala1-3Galb1-3GlcNAcb1-2Mana1-3)Manb1-4GlcNAcb1-4GlcNAc-Sp19				
**383**	Gala1-3Galb1-3(Fuca1-4)GlcNAcb1-2Mana1-6(Gala1-3Galb1-3(Fuca1-4)GlcNAcb1-2Mana1-3)Manb1-4GlcNAcb1-4GlcNAc-Sp19				
**384**	GlcNAcb1-2Mana1-6(Galb1-4GlcNAcb1-2Mana1-3)Manb1-4GlcNAcb1-4GlcNAc-Sp12		9.27		
**385**	Galb1-4GlcNAcb1-2Mana1-6(GlcNAcb1-2Mana1-3)Manb1-4GlcNAcb1-4GlcNAc-Sp12				
**386**	Neu5Aca2-3Galb1-3GlcNAcb1-3GalNAca-Sp14	2.6			
**387**	Fuca1-2Galb1-4GlcNAcb1-3GalNAca-Sp14		12.52		
**388**	Galb1-4(Fuca1-3)GlcNAcb1-3GalNAca-Sp14				
**389**	GalNAca1-3GalNAcb1-3Gala1-4Galb1-4GlcNAcb-Sp0				
**390**	Gala1-4Galb1-3GlcNAcb1-2Mana1-6(Gala1-4Galb1-3GlcNAcb1-2Mana1-3)Manb1-4GlcNAcb1-4GlcNAcb-Sp19				

**391**	Gala1-4Galb1-4GlcNAcb1-2Mana1-6(Gala1-4Galb1-4GlcNAcb1-2Mana1-3)Manb1-4GlcNAcb1-4GlcNAcb-Sp24				
**392**	Gala1-3Galb1-4GlcNAcb1-3GalNAca-Sp14		41.34		19.89
**393**	Galb1-3GlcNAcb1-6Galb1-4GlcNAcb-Sp0				
**394**	Galb1-3GlcNAca1-6Galb1-4GlcNAcb-Sp0				
**395**	GalNAcb1-3Gala1-6Galb1-4Glcb-Sp8				
**396**	Gala1-3(Fuca1-2)Galb1-4(Fuca1-3)Glcb-Sp21				
**397**	Galb1-4GlcNAcb1-6(Neu5Aca2-6Galb1-3GlcNAcb1-3)Galb1-4Glc-Sp21				
**398**	Galb1-3GalNAcb1-4(Neu5Aca2-8Neu5Aca2-3)Galb1-4Glcb-Sp0				
**399**	Neu5Aca2-3Galb1-3GalNAcb1-4(Neu5Aca2-8Neu5Aca2-3)Galb1-4Glcb-Sp0				
**400**	Gala1-3(Fuca1-2)Galb1-4GlcNAcb1-3GalNAca-Sp14	1.2			59.42
**401**	GalNAca1-3(Fuca1-2)Galb1-4GlcNAcb1-3GalNAca-Sp14	3.4			23.33
**402**	GalNAca1-3GalNAcb1-3Gala1-4Galb1-4Glcb-Sp0				
**403**	Fuca1-2Galb1-4(Fuca1-3)GlcNAcb1-3GalNAca-Sp14				
**404**	Gala1-3(Fuca1-2)Galb1-4(Fuca1-3)GlcNAcb1-3GalNAc-Sp14				
**405**	GalNAca1-3(Fuca1-2)Galb1-4(Fuca1-3)GlcNAcb1-3GalNAc-Sp14				
**406**	Galb1-4(Fuca1-3)GlcNAcb1-2Mana1-6(Galb1-4(Fuca1-3)GlcNAcb1-2Mana1-3)Manb1-4GlcNAcb1-4(Fuca1-6)GlcNAcb-Sp22				
**407**	Fuca1-2Galb1-4GlcNAcb1-2Mana1-6(Fuca1-2Galb1-4GlcNAcb1-2Mana1-3)Manb1-4GlcNAcb1-4(Fuca1-6)GlcNAcb-Sp22		10.39		
**408**	GlcNAcb1-2(GlcNAcb1-6)Mana1-6(GlcNAcb1-2Mana1-3)Manb1-4GlcNAcb1-4GlcNAcb-Sp19				
**409**	Fuca1-2Galb1-3GlcNAcb1-3GalNAc-Sp14		21.44		11.19
**410**	Gala1-3(Fuca1-2)Galb1-3GlcNAcb1-3GalNAc-Sp14	2.0			39.44
**411**	GalNAca1-3(Fuca1-2)Galb1-3GlcNAcb1-3GalNAc-Sp14		10.09		
**412**	Gala1-3Galb1-3GlcNAcb1-3GalNAc-Sp14		23.51		9.62
**413**	Fuca1-2Galb1-3GlcNAcb1-2Mana1-6(Fuca1-2Galb1-3GlcNAcb1-2Mana1-3)Manb1-4GlcNAcb1-4(Fuca1-6)GlcNAcb-Sp22				
**414**	Gala1-3(Fuca1-2)Galb1-4GlcNAcb1-2Mana1-6(Gala1-3(Fuca1-2)Galb1-4GlcNAcb1-2Mana1-3)Manb1-4GlcNAcb1-4(Fuca1-6)GlcNAcb-Sp22	1.2			50.22
**415**	Galb1-3GlcNAcb1-6(Galb1-3GlcNAcb1-2)Mana1-6(Galb1-3GlcNAcb1-2Mana1-3)Manb1-4GlcNAcb1-4GlcNAcb-Sp19		10.15		
**416**	Galb1-4GlcNAcb1-6(Fuca1-2Galb1-3GlcNAcb1-3)Galb1-4Glc-Sp21		34.81		13.12
**417**	Fuca1-3GlcNAcb1-6(Galb1-4GlcNAcb1-3)Galb1-4Glc-Sp21				
**418**	GlcNAcb1-2Mana1-6(GlcNAcb1-4)(GlcNAcb1-2Mana1-3)Manb1-4GlcNAcb1-4GlcNAc-Sp21				
**419**	GlcNAcb1-2Mana1-6(GlcNAcb1-4)(GlcNAcb1-4(GlcNAcb1-2)Mana1-3)Manb1-4GlcNAcb1-4GlcNAc-Sp21				
**420**	GlcNAcb1-6(GlcNAcb1-2)Mana1-6(GlcNAcb1-4)(GlcNAcb1-2Mana1-3)Manb1-4GlcNAcb1-4GlcNAc-Sp21				
**421**	GlcNAcb1-6(GlcNAcb1-2)Mana1-6(GlcNAcb1-4)(GlcNAcb1-4(GlcNAcb1-2)Mana1-3)Manb1-4GlcNAcb1-4GlcNAc-Sp21				
**422**	Galb1-4GlcNAcb1-2Mana1-6(GlcNAcb1-4)(Galb1-4GlcNAcb1-2Mana1-3)Manb1-4GlcNAcb1-4GlcNAc-Sp21				
**423**	Galb1-4GlcNAcb1-2Mana1-6(GlcNAcb1-4)(Galb1-4GlcNAcb1-4(Galb1-4GlcNAcb1-2)Mana1-3)Manb1-4GlcNAcb1-4GlcNAc-Sp21		7.55		
**424**	Galb1-4GlcNAcb1-6(Galb1-4GlcNAcb1-2)Mana1-6(GlcNAcb1-4)(Galb1-4GlcNAcb1-2Mana1-3)Manb1-4GlcNAcb1-4GlcNAc-Sp21		8.41		
**425**	Galb1-4GlcNAcb1-6(Galb1-4GlcNAcb1-2)Mana1-6(GlcNAcb1-4)(Galb1-4GlcNAcb1-4(Galb1-4GlcNAcb1-2)Mana1-3)Manb1-4GlcNAcb1-4GlcNAc-Sp21	8.0			7.47
**426**	Galb1-4Galb-Sp10				
**427**	Galb1-6Galb-Sp10				
**428**	Neu5Aca2-3Galb1-4GlcNAcb1-3Galb-Sp8		12.06		
**429**	GalNAcb1-6GalNAcb-Sp8				
**430**	(6S)Galb1-3GlcNAcb-Sp0				
**431**	(6S)Galb1-3(6S)GlcNAc-Sp0				
**432**	Fuca1-2Galb1-4 GlcNAcb1-2Mana1-6(Fuca1-2Galb1-4GlcNAcb1-2(Fuca1-2Galb1-4GlcNAcb1-4)Mana1-3)Manb1-4GlcNAcb1-4GlcNAcb-Sp12		23.91		21.22
**433**	Fuca1-2Galb1-4(Fuca1-3)GlcNAcb1-2Mana1-6(Fuca1-2Galb1-4(Fuca1-3)GlcNAcb1-4(Fuca1-2Galb1-4(Fuca1-3)GlcNAcb1-2)Mana1-3)Manb1-4GlcNAcb1-4GlcNAcb-Sp12				
**434**	Galb1-4(Fuca1-3)GlcNAcb1-6GalNAc-Sp14				

**435**	Galb1-4GlcNAcb1-2Mana-Sp0				
**436**	Fuca1-2Galb1-4GlcNAcb1-6(Fuca1-2Galb1-4GlcNAcb1-3)GalNAc-Sp14		15.13		8.85
**437**	Gala1-3(Fuca1-2)Galb1-4GlcNAcb1-6(Gala1-3(Fuca1-2)Galb1-4GlcNAcb1-3)GalNAc-Sp14	1.3		7.5	
**438**	GalNAca1-3(Fuca1-2)Galb1-4GlcNAcb1-6(GalNAca1-3(Fuca1-2)Galb1-4GlcNAcb1-3)GalNAc-Sp14	2.2			47.9
**439**	Neu5Aca2-8Neu5Aca2-3Galb1-3GalNAcb1-4(Neu5Aca2-8Neu5Aca2-3)Galb1-4Glcb-Sp0		22.7		9.26
**440**	GalNAcb1-4Galb1-4Glcb-Sp0				
**441**	GalNAca1-3(Fuca1-2)Galb1-4GlcNAcb1-2Mana1-6(GalNAca1-3(Fuca1-2)Galb1-4GlcNAcb1-2Mana1-3)Manb1-4GlcNAcb1-4(Fuca1-6)GlcNAcb-Sp22		15.98		8.96
**442**	Gala1-3(Fuca1-2)Galb1-3GlcNAcb1-2Mana1-6(Gala1-3(Fuca1-2)Galb1-3GlcNAcb1-2Mana1-3)Manb1-4GlcNAcb1-4(Fuca1-6)GlcNAcb-Sp22		21.75		23.64
**443**	Neu5Aca2-6Galb1-4GlcNAcb1-6(Fuca1-2Galb1-3GlcNAcb1-3)Galb1-4Glc-Sp21				
**444**	GalNAca1-3(Fuca1-2)Galb1-3GlcNAcb1-2Mana1-6(GalNAca1-3(Fuca1-2)Galb1-3GlcNAcb1-2Mana1-3)Manb1-4GlcNAcb1-4(Fuca1-6)GlcNAcb-Sp22		13.48		11.74
**445**	Galb1-4GlcNAcb1-6(Galb1-4GlcNAcb1-2)Mana1-6(Galb1-4GlcNAcb1-2Mana1-3)Manb1-4GlcNAcb1-4GlcNAcb-Sp19		9.39		11.11
**446**	Neu5Aca2-3Galb1-4GlcNAcb1-4Mana1-6(GlcNAcb1-4)(Neu5Aca2-3Galb1-4GlcNAcb1-4(Neu5Aca2-3Galb1-4GlcNAcb1-2)Mana1-3)Manb1-4GlcNAcb1-4GlcNAcb-Sp21		32.72		25.78
**447**	Neu5Aca2-3Galb1-4GlcNAcb1-6(Neu5Aca2-3Galb1-4GlcNAcb1-2)Mana1-6(GlcNAcb1-4)(Neu5Aca2-3Galb1-4GlcNAcb1-2Mana1-3)Manb1-4GlcNAcb1-4GlcNAcb-Sp21		40.83		12.19
**448**	Neu5Aca2-3Galb1-4GlcNAcb1-6(Neu5Aca2-3Galb1-4GlcNAcb1-2)Mana1-6(GlcNAcb1-4)(Neu5Aca2-3Galb1-4GlcNAcb1-4(Neu5Aca2-3Galb1-4GlcNAcb1-2)Mana1-3)Manb1-4GlcNAcb1-4GlcNAcb-Sp21	7.8			19.64
**449**	Neu5Aca2-6Galb1-4GlcNAcb1-4Mana1-6(GlcNAcb1-4)(Neu5Aca2-6Galb1-4GlcNAcb1-4(Neu5Aca2-6Galb1-4GlcNAcb1-2)Mana1-3)Manb1-4GlcNAcb1-4GlcNAcb-Sp21				
**450**	Neu5Aca2-6Galb1-4GlcNAcb1-6(Neu5Aca2-6Galb1-4GlcNAcb1-2)Mana1-6(GlcNAcb1-4)(Neu5Aca2-6Galb1-4GlcNAcb1-2Mana1-3)Manb1-4GlcNAcb1-4GlcNAcb-Sp21				
**451**	Gala1-3(Fuca1-2)Galb1-3GalNAca-Sp8				
**452**	Gala1-3(Fuca1-2)Galb1-3GalNAcb-Sp8				
**453**	Glca1-6Glca1-6Glca1-6Glcb-Sp10				
**454**	Glca1-4Glca1-4Glca1-4Glcb-Sp10				
**455**	Neu5Aca2-3Galb1-4GlcNAcb1-6(Neu5Aca2-3Galb1-4GlcNAcb1-3)GalNAca-Sp14		8.84		
**456**	Fuca1-2Galb1-4(Fuca1-3)GlcNAcb1-2Mana1-6(Fuca1-2Galb1-4(Fuca1-3)GlcNAcb1-2Mana1-3)Manb1-4GlcNAcb1-4(Fuca1-6)GlcNAcb-Sp24				
**457**	Fuca1-2Galb1-3(Fuca1-4)GlcNAcb1-2Mana1-6(Fuca1-2Galb1-3(Fuca1-4)GlcNAcb1-2Mana1-3)Manb1-4GlcNAcb1-4(Fuca1-6)GlcNAcb1-4(Fuca1-6)GlcNAcb-Sp19				
**458**	GlcNAcb1-6(GlcNAcb1-2)Mana1-6(GlcNAcb1-2Mana1-3)Manb1-4GlcNAcb1-4(Fuca1-6)GlcNAcb-Sp24				
**459**	Galb1-3GlcNAcb1-2Mana1-6(GlcNAcb1-4)(Galb1-3GlcNAcb1-2Mana1-3)Manb1-4GlcNAcb1-4GlcNAcb-Sp21				
**460**	Neu5Aca2-6Galb1-4GlcNAcb1-6(Galb1-3GlcNAcb1-3)Galb1-4Glcb-Sp21				
**461**	Neu5Aca2-3Galb1-4GlcNAcb1-2Mana-Sp0				
**462**	Neu5Aca2-3Galb1-4GlcNAcb1-6GalNAca-Sp14		7.88		
**463**	Neu5Aca2-6Galb1-4GlcNAcb1-6GalNAca-Sp14				
**464**	Neu5Aca2-6Galb1-4 GlcNAcb1-6(Neu5Aca2-6Galb1-4GlcNAcb1-3)GalNAca-Sp14				
**465**	Neu5Aca2-6Galb1-4GlcNAcb1-2Mana1-6(Neu5Aca2-6Galb1-4GlcNAcb1-2Mana1-3)Manb1-4GlcNAcb1-4(Fuca1-6)GlcNAcb-Sp24				
**466**	Neu5Aca2-3Galb1-4GlcNAcb1-2Mana1-6(Neu5Aca2-3Galb1-4GlcNAcb1-2Mana1-3)Manb1-4GlcNAcb1-4(Fuca1-6)GlcNAcb-Sp24		30.67		14.35
**467**	Mana1-6(Mana1-3)Manb1-4GlcNAcb1-4(Fuca1-6)GlcNAcb-Sp19				
**468**	Galb1-4GlcNAcb1-6(Galb1-4GlcNAcb1-2)Mana1-6(Galb1-4GlcNAcb1-2Mana1-3)Manb1-4GlcNAcb1-4(Fuca1-6)GlcNAcb-Sp24		28.83		12.68

**469**	Neu5Aca2-3Galb1-3GlcNAcb1-2Mana1-6(GlcNAcb1-4)(Neu5Aca2-3Galb1-3GlcNAcb1-2Mana1-3)Manb1-4GlcNAcb1-4GlcNAc-Sp21				
**470**	Neu5Aca2-6Galb1-4GlcNAcb1-6(Fuca1-2Galb1-4(Fuca1-3)GlcNAcb1-3)Galb1-4Glc-Sp21				
**471**	Galb1-3GlcNAcb1-6GalNAca-Sp14				
**472**	Gala1-3Galb1-3GlcNAcb1-6GalNAca-Sp14		20.18		9.97
**473**	Galb1-3(Fuca1-4)GlcNAcb1-6GalNAca-Sp14				
**474**	Neu5Aca2-3Galb1-3GlcNAcb1-6GalNAca-Sp14				
**475**	(3S)Galb1-3(Fuca1-4)GlcNAcb-Sp0				
**476**	Galb1-4(Fuca1-3)GlcNAcb1-6(Neu5Aca2-6(Neu5Aca2-3Galb1-3)GlcNAcb1-3)Galb1-4Glc-Sp21				
**477**	Fuca1-2Galb1-4GlcNAcb1-6GalNAca-Sp14		9.04		
**478**	Gala1-3Galb1-4GlcNAcb1-6GalNAca-Sp14		30.48		14.61
**479**	Galb1-4(Fuca1-3)GlcNAcb1-2Mana-Sp0				
**480**	Fuca1-2(6S)Galb1-3GlcNAcb-Sp0				
**481**	Gala1-3(Fuca1-2)Galb1-4GlcNAcb1-6GalNAca-Sp14				
**482**	Fuca1-2Galb1-4GlcNAcb1-2Mana-Sp0				
**483**	Fuca1-2Galb1-3(6S)GlcNAcb-Sp0		10.88		
**484**	Fuca1-2(6S)Galb1-3(6S)GlcNAcb-Sp0				
**485**	Neu5Aca2-6GalNAcb1-4(6S)GlcNAcb-Sp8				
**486**	GalNAcb1-4(Fuca1-3)(6S)GlcNAcb-Sp8				
**487**	(3S)GalNAcb1-4(Fuca1-3)GlcNAcb-Sp8				
**488**	Fuca1-2Galb1-3GlcNAcb1-6(Fuca1-2Galb1-3GlcNAcb1-3)GalNAca-Sp14				
**489**	GalNAca1-3(Fuca1-2)Galb1-3GlcNAcb1-6GalNAca-Sp14		44.38		27.1
**490**	GlcNAcb1-6(GlcNAcb1-2)Mana1-6(GlcNAcb1-4)(GlcNAcb1-4(GlcNAcb1-2)Mana1-3)Manb1-4GlcNAcb1-4(Fuca1-6)GlcNAc-Sp21				
**491**	Galb1-4GlcNAcb1-6(Galb1-4GlcNAcb1-2)Mana1-6(GlcNAcb1-4)Galb1-4GlcNAcb1-4(Gal b1-4GlcNAcb1-2)Mana1-3)Manb1-4GlcNAcb1-4(Fuca1-6)GlcNAc-Sp21		52.67		50.85
**492**	Galb1-3GlcNAca1-3Galb1-4GlcNAcb-Sp8		9.77		
**493**	Galb1-3(6S)GlcNAcb-Sp8				
**494**	(6S)(4S)GalNAcb1-4GlcNAc-Sp8				
**495**	(6S)GalNAcb1-4GlcNAc-Sp8				
**496**	(3S)GalNAcb1-4(3S)GlcNAc-Sp8				
**497**	GalNAcb1-4(6S)GlcNAc-Sp8				
**498**	(3S)GalNAcb1-4GlcNAc-Sp8		21.58		16.61
**499**	(4S)GalNAcb-Sp10				
**500**	Galb1-4(6P)GlcNAcb-Sp0				
**501**	(6P)Galb1-4GlcNAcb-SP0				
**502**	GalNAca1-3(Fuca1-2)Galb1-4GlcNAcb1-6GalNAc-Sp14	3.2			35.57
**503**	Neu5Aca2-6Galb1-4GlcNAcb1-2Man-Sp0				
**504**	Gala1-3Galb1-4GlcNAcb1-2Mana-Sp0	2.4			29.76
**505**	GalNAca1-3(Fuca1-2)Galb1-4 GlcNAcb1-2Mana-Sp0	1.1			66.69
**506**	Galb1-3GlcNAcb1-2Mana-Sp0				
**507**	Gala1-3(Fuca1-2)Galb1-3GlcNAcb1-6GalNAc-Sp14	1.8			36.52
**508**	Neu5Aca2-3Galb1-3GlcNAcb1-2Mana-Sp0		9.64		
**509**	Gala1-3Galb1-3GlcNAcb1-2Mana-Sp0	9.7			17.93
**510**	GalNAcb1-4GlcNAcb1-2Mana-Sp0				
**511**	Neu5Aca2-3Galb1-3GalNAcb1-4Galb1-4Glcb-Sp0				
**512**	GlcNAcb1-2 Mana1-6(GlcNAcb1-4)(GlcNAcb1-2Mana1-3)Manb1-4GlcNAcb1-4(Fuca1-6)GlcNAc-Sp21				
**513**	Galb1-4GlcNAcb1-2 Mana1-6(GlcNAcb1-4)(Galb1-4GlcNAcb1-2Mana1-3)Manb1-4GlcNAcb1-4(Fuca1-6)GlcNAc-Sp21		23.18		9.29
**514**	Galb1-4GlcNAcb1-2 Mana1-6(Galb1-4GlcNAcb1-4)(Galb1-4GlcNAcb1-2Mana1-3)Manb1-4GlcNAcb1-4(Fuca1-6)GlcNAc-Sp21				
**515**	Fuca1-4(Galb1-3)GlcNAcb1-2 Mana-Sp0				
**516**	Neu5Aca2-3Galb1-4(Fuca1-3)GlcNAcb1-2Mana-Sp0				
**517**	GlcNAcb1-3Galb1-4GlcNAcb1-6(GlcNAcb1-3)Galb1-4GlcNAc-Sp0	9.8			9.93
**518**	GalNAca1-3(Fuca1-2)Galb1-3GalNAcb1-3Gala1-4Galb1-4Glc-Sp21				
**519**	Gala1-3(Fuca1-2)Galb1-3GalNAcb1-3Gala1-4Galb1-4Glc-Sp21				
**520**	Galb1-3GalNAcb1-3Gal-Sp21				
**521**	GlcNAcb1-3Galb1-4GlcNAcb1-2Mana1-6(GlcNAcb1-3Galb1-4GlcNAcb1-2Mana1-3)Manb1-4GlcNAcb1-4GlcNAcb-Sp12				
**522**	Galb1-4GlcNAcb1-3Galb1-4GlcNAcb1-2Mana1-6(Galb1-4GlcNAcb1-3Galb1-4GlcNAcb1-2Mana1-3)Manb1-4GlcNAcb1-4GlcNAcb-Sp12				

**523**	GlcNAcb1-3Galb1-4GlcNAcb1-3Galb1-4GlcNAcb1-2Mana1-6(GlcNAcb1-3Galb1-4GlcNAcb1-3Galb1-4GlcNAcb1-2Mana1-3)Manb1-4GlcNAcb1-4GlcNAcb-Sp12	5.0			23.19
**524**	Galb1-4GlcNAcb1-3Galb1-4GlcNAcb1-3Galb1-4GlcNAcb1-2Mana1-6(Galb1-4GlcNAcb1-3Galb1-4GlcNAcb1-3Galb1-4GlcNAcb1-2Mana1-3)Manb1-4GlcNAcb1-4GlcNAcb-Sp12	2.4			56.13
**525**	Galb1-3GlcNAcb1-3Galb1-4GlcNAcb1-2Mana1-6(Galb1-3GlcNAcb1-3Galb1-4GlcNAcb1-2Mana1-3)Manb1-4GlcNAcb1-4GlcNAc-Sp25	5.5			33.12
**526**	Neu5Gca2-8Neu5Gca2-3Galb1-4GlcNAc-Sp0		28.94		9.24
**527**	Neu5Aca2-8Neu5Gca2-3Galb1-4GlcNAc-Sp0	1.7			7.63
**528**	Neu5Gca2-8Neu5Aca2-3Galb1-4GlcNAc-Sp0		18.47		
**529**	Neu5Gca2-8Neu5Gca2-3Galb1-4GlcNAcb1-3Galb1-4GlcNAc-Sp0		27.73		24.99
**530**	Neu5Gca2-8Neu5Gca2-6Galb1-4GlcNAc-Sp0				
**531**	Neu5Aca2-8Neu5Aca2-3Galb1-4GlcNAc-Sp0	3.3			
**532**	GlcNAcb1-3Galb1-4GlcNAcb1-6(GlcNAcb1-3Galb1-4GlcNAcb1-2)Mana1-6(GlcNAcb1-3Galb1-4GlcNAcb1-2Man a1-3)Manb1-4GlcNAcb1-4GlcNAc-Sp24	6.3			40.29
**533**	Galb1-4GlcNAcb1-3Galb1-4GlcNAcb1-6(Galb1-4GlcNAcb1-3Galb1-4GlcNAcb1-2)Mana1-6(Galb1-4GlcNAcb1-3Galb1-4GlcNAcb1-2Mana1-3)Mana1-4GlcNAcb1-4GlcNAc-Sp24	6.8			23.76
**534**	Gala1-3Galb1-4GlcNAcb1-2Mana1-6(Gala1-3Galb1-4GlcNAcb1-2Mana1-3)Manb1-4GlcNAcb1-4GlcNAc-Sp24	2.5			75.87
**535**	GlcNAcb1-3Galb1-4GlcNAcb1-6(GlcNAcb1-3Galb1-3)GalNAca-Sp14		11.36		
**536**	GalNAcb1-3GlcNAcb-Sp0		8.13		
**537**	GalNAcb1-4GlcNAcb1-3GalNAcb1-4GlcNAcb-Sp0	2.2			69.51
**538**	GlcNAb1-3Galb1-3GalNAc-Sp14				
**539**	Galb1-3GlcNAcb1-6(Galb1-3)GalNAc-Sp14		6.51		
**540**	(3S)GlcAb1-3Galb1-4GlcNAcb1-3Galb1-4Glc-Sp0	1.7			50.79
**541**	(3S)GlcAb1-3Galb1-4GlcNAcb1-2Mana-Sp0	2.6			40.35
**542**	Galb1-3GlcNAcb1-3Galb1-4GlcNAcb1-3Galb1-4GlcNAcb1-6(Galb1-3GlcNAcb1-3Galb1-4GlcNAcb1-3Galb1-4GlcNAb1-2)Mana1-6(Galb1-3GlcNAcb1-3Galb1-4GlcNAcb1-3Galb1-4GlcNAcb1-2Mana1-3)Manb1-4GlcNAcb1-4(Fuca1-6)GlcNAcb-Sp24	3.1			46.35
**543**	Galb1-3GlcNAcb1-3Galb1-4GlcNAcb1-6(Galb1-3GlcNAcb1-3Galb1-4GlcNAb1-2)Mana1-6(Galb1-3GlcNAcb1-3Galb1-4GlcNAcb1-2Mana1-3)Manb1-4GlcNAcb1-4(Fuca1-6)GlcNAcb-Sp24				
**544**	Galb1-4GlcNAcb1-3Galb1-4GlcNAcb1-3GalNAca-Sp14	8.2			17.9
**545**	Galb1-4GlcNAcb1-3Galb1-4GlcNAcb1-6(Galb1-3)GalNAca-Sp14		32.41		18.95
**546**	Galb1-4GlcNAcb1-3Galb1-4GlcNAcb1-6(Galb1-4GlcNAcb1-3Galb1-4GlcNAcb1-3)GalNAca-Sp14	9.0			34.09
**547**	GlcNAcb1-3Galb1-4GlcNAcb1-3GalNAca-Sp14		19.87		15.52
**548**	GlcNAcb1-3Galb1-4GlcNAcb1-6(Galb1-3)GalNAca-Sp14		17.04		11.4
**549**	GlcNAcb1-3Galb1-4GlcNAcb1-6(GlcNAcb1-3Galb1-4GlcNAcb1-3)GalNAca-Sp14	8.5			27.99
**550**	GlcNAcb1-3Galb1-4GlcNAcb1-3Galb1-4GlcNAcb1-3GalNAca-Sp14	4.7			38.89
**551**	Galb1-4GlcNAcb1-3Galb1-3GalNAca-Sp14				
**552**	Neu5Aca2-6Galb1-4GlcNAcb1-6(Galb1-3)GalNAca-Sp14				
**553**	GlcNAcb1-6(Neu5Aca2-3Galb1-3)GalNAca-Sp14				
**554**	Galb1-3GalNAcb1-4(Neu5Aca2-8Neu5Aca2-8Neu5Aca2-3)Galb1-4Glcb-Sp21				

Supplementary Table 1. Gal-3 and Gal-3C binding to glycans present on the CFG microarray. Each glycan is numbered, with each structure listed and the apparent K_D value or % max binding at the highest concentration tested for glycan binding that did not result in saturation over the concentrations tested. Gal-3: galectin-3. Gal-3C: the C-terminal domain of galectin-3.
