# Supplementary material for: Full-Length Galectin-3 Is Required for High Affinity Microbial Interactions and Antimicrobial Activity
Source: Front Microbiol. 2021 Oct 8;12:731026. doi: 10.3389/fmicb.2021.731026 (PMC8531552; doi:10.3389/fmicb.2021.731026)
Supplement: Supplementary file 4 [file Data_Sheet_4.PDF]

Wu et al. Supplementary Table 2 (MGM).

Glycan Number	Bacteria Strain	Structure Cat. No.	Gal-3 K_D	Gal-3 % max	Gal-3C K_D	Gal-3C % max
**313**	1-2 Mannan Acetobacter methanolieus MB135					
**307**	Davanat					
**305**	Escherichia coli K13	CPS				
**18**	Escherichia coli K235 LPS	L2143				
**304**	Escherichia coli K5	CPS				
**303**	Escherichia coli K92	CPS				
**32**	Escherichia coli O106					
**298**	Escherichia coli O107	PS				
**222**	Escherichia coli O111:B4 LPS- solution at 1 mg/mL	L5293-2ML (LPS) (Sigma)				
**210**	Escherichia coli O112ab	OPS				
**211**	Escherichia coli O118	OPS				
**212**	Escherichia coli O125	OPS				
**225**	Escherichia coli O127:B8 LPS- solution at 1 mg/mL	L5668-2ML (LPS) (Sigma)				
**19**	Escherichia coli O128-B12 LPS	L2755				
**33**	Escherichia coli O130					
**297**	Escherichia coli O145	LPSOH				
**34**	Escherichia coli O148					
**35**	Escherichia coli O150					
**213**	Escherichia coli O151	OPS				
**214**	Escherichia coli O168	OPS				
**36**	Escherichia coli O180		4.9			
**223**	Escherichia coli O26:B6 LPS- solution at 1 mg/mL	L5543-2ML (LPS) (Sigma)				
**30**	Escherichia coli O29					
**31**	Escherichia coli O40					
**205**	Escherichia coli O49	OPS				
**206**	Escherichia coli O52	OPS				
**224**	Escherichia coli O55:B5 LPS- solution at 1 mg/mL	L5418-2ML (LPS) (Sigma)	4.5			18.51
**207**	Escherichia coli O58	OPS				
**208**	Escherichia coli O61	LPSOH				
**294**	Escherichia coli O71	PS				
**209**	Escherichia coli O73	OPS				
**295**	Escherichia coli O85	PS				
**296**	Escherichia coli O99	PS				
**185**	Francisella novicida OPS	OPS				
**186**	Francisella tularensis OPS	OPS				
**311**	Galactomannan DAVANT (160102) Pro-Pharmacenti					
**187**	Klebsiella O1 OPS	OPS		49.68		24.0
**194**	Klebsiella O12 OPS	OPS				
**188**	Klebsiella O2a OPS	OPS		29.61		
**189**	Klebsiella O2ac OPS	OPS		33.38		
**190**	Klebsiella O3 OPS	OPS				
**191**	Klebsiella O4 OPS	OPS				
**192**	Klebsiella O5 OPS	OPS				
**193**	Klebsiella O8 OPS	OPS	5.9			85.23
**308**	Laminarin					
**306**	Neisseria meningitidis Group C	CPS				
**39**	Proteus mirabilis O10 (HJ4320)					
**264**	Proteus mirabilis O11 (9B-m)	PS				
**92**	Proteus mirabilis O11 (PrK 24/57)	OPS				
**93**	Proteus mirabilis O13 (PrK 26/57)	OPS				
**94**	Proteus mirabilis O14a,14b (PrK 29/57)	OPS				
**95**	Proteus mirabilis O16 (4652)	OPS				
**96**	Proteus mirabilis O17 (PrK 32/57)	OPS				
**266**	Proteus mirabilis O18 (PrK 34/57)	LPSOH				
**267**	Proteus mirabilis O20 (PrK 38/57)	LPSOH				
**174**	Proteus mirabilis O23a, 23b, 23c (CCUG 10701)	OPS				
**97**	Proteus mirabilis O23a,b,d (PrK 42/57)	OPS				
**98**	Proteus mirabilis O26 (PrK 49/57)	OPS				
**99**	Proteus mirabilis O27 (PrK 50/57)	OPS			3.2	
**100**	Proteus mirabilis O28 (PrK 51/57)	OPS				
**101**	Proteus mirabilis O29a (PrK 52/57)	OPS				
**40**	Proteus mirabilis O29a, 29b (2002)					
**269**	Proteus mirabilis O33 (D52)	PS		52.01		15.69
**37**	Proteus mirabilis O3a, 3c (G1)					
**261**	Proteus mirabilis O3ab (S1959)	PS				
**102**	Proteus mirabilis O40 (10703)	OPS				
**103**	Proteus mirabilis O41 (PrK 67/57)	OPS				
**270**	Proteus mirabilis O43 (PrK 69/57)	PS				
**272**	Proteus mirabilis O49 (PrK 75/57)	PS	5.4			
**262**	Proteus mirabilis O5 (PrK 12/57)	PS				
**41**	Proteus mirabilis O50 (TG332)					
**104**	Proteus mirabilis O51 (19011)*	LPS				
**42**	Proteus mirabilis O54a, 54b (10704)					
**273**	Proteus mirabilis O54ab (OE)	PS				
**43**	Proteus mirabilis O57 (TG319)					

**91**	Proteus mirabilis O6 (PrK 14/57)	OPS				
**105**	Proteus mirabilis O74 (10705, OF)	OPS				
**106**	Proteus mirabilis O75 (10702, OC)	OPS				
**107**	Proteus mirabilis O77 (3 B-m)	OPS				
**38**	Proteus mirabilis O8 (TG326)					
**263**	Proteus mirabilis O9 (PrK 18/57)	PS				
**121**	Proteus myxofaciens O60	OPS				
**122**	Proteus O56 (genomospecies 4)	OPS				
**265**	Proteus penneri O17 (16)	PS				
**108**	Proteus penneri O31a (26)	OPS				
**268**	Proteus penneri O31ab (28)	PS				
**109**	Proteus penneri O52 (15)	OPS				
**110**	Proteus penneri O58 (12)	OPS				
**111**	Proteus penneri O59 (9)	OPS				
**112**	Proteus penneri O61 (21)	OPS				
**113**	Proteus penneri O62 (41)	OPS				
**114**	Proteus penneri O63 (22)	OPS				
**45**	Proteus penneri O64a, 64b, 64d (39)					
**115**	Proteus penneri O64a,b,c (27)	OPS				
**116**	Proteus penneri O65 (34)	OPS				
**46**	Proteus penneri O66 (2)					
**117**	Proteus penneri O67 (8)	OPS				
**118**	Proteus penneri O68 (63)	OPS				
**47**	Proteus penneri O69 (25)					
**119**	Proteus penneri O70 (60)	OPS				
**48**	Proteus penneri O71 (42)					
**49**	Proteus penneri O72a, 72b (4)					
**120**	Proteus penneri O73a,b (103)	OPS				
**274**	Proteus penneri O73ac (75)	PS		4.39		
**44**	Proteus penneri O8 (106)					
**72**	Proteus vulgaris O1 (18984)*	LPS				
**74**	Proteus vulgaris O12 (PrK 25/57)	OPS				
**75**	Proteus vulgaris O13 (8344)	OPS				
**76**	Proteus vulgaris O15 (PrK 30/57)	OPS				
**77**	Proteus vulgaris O17 (PrK 33/57)	OPS				
**78**	Proteus vulgaris O19a (PrK 37/57)	OPS				
**260**	Proteus vulgaris O2 (OX2)	PS				
**79**	Proteus vulgaris O21 (PrK 39/57)*	LPS				
**80**	Proteus vulgaris O22 (PrK 40/57)	OPS		8.5		
**175**	Proteus vulgaris O24 (PrK 47/57)	LPSOH				
**81**	Proteus vulgaris O25 (PrK 48/57)	OPS		6.53		
**82**	Proteus vulgaris O34 (4669)*	LPS				
**83**	Proteus vulgaris O37a,b (PrK 63/57)	OPS				
**84**	Proteus vulgaris O37a,c (PrK 72/57)	OPS	5.1			
**73**	Proteus vulgaris O4 (PrK 9/57)	OPS				
**85**	Proteus vulgaris O44 (PrK 67/57)	OPS				
**86**	Proteus vulgaris O45 (4680)	OPS	7.0			25.84
**271**	Proteus vulgaris O47 (PrK 73/57)	Not stated	1.8			72.03
**87**	Proteus vulgaris O53 (TG 276-10)	OPS				
**88**	Proteus vulgaris O54a,54c (TG 103)	OPS				
**89**	Proteus vulgaris O55 (TG 155)	OPS				
**90**	Proteus vulgaris O65 (TG 251)	OPS				
**275**	Proteus vulgaris O76 (HSC438)	PS				
**134**	Providencia alcalifaciens O19	OPS				
**135**	Providencia alcalifaciens O19	LPS				
**136**	Providencia alcalifaciens O19	LPS/NaOH				
**137**	Providencia alcalifaciens O21	OPS				
**138**	Providencia alcalifaciens O23	OPS				
**139**	Providencia alcalifaciens O27	OPS				
**140**	Providencia alcalifaciens O29	OPS				
**141**	Providencia alcalifaciens O30	OPS				
**142**	Providencia alcalifaciens O32	OPS				
**143**	Providencia alcalifaciens O36*	LPS-NH4OH				
**144**	Providencia alcalifaciens O39	OPS				
**132**	Providencia alcalifaciens O5	OPS	0.7		4.2	
**133**	Providencia alcalifaciens O6*	LPS	2.6			56.15
**145**	Providencia rustigianii O14	OPS				
**146**	Providencia rustigianii O16	OPS				
**147**	Providencia rustigianii O34	OPS				
**124**	Providencia stuartii O18	OPS				
**125**	Providencia stuartii O20*	LPS				
**123**	Providencia stuartii O4	OPS				
**126**	Providencia stuartii O43	OPS				
**127**	Providencia stuartii O44	OPS				
**128**	Providencia stuartii O47	OPS				
**129**	Providencia stuartii O47, Core 9	OPS				

**1**	Providencia stuartii O49	PO49 Core-linked				
**130**	Providencia stuartii O49, Core 1	OPS				
**2**	Providencia stuartii O52	PO52 Core-linked				
**131**	Providencia stuartii O57	OPS	3.2			
**4**	Pseudomonas aerguinosa O1 (Fisher immunotype 4)	PO1 Core-linked				
**13**	Pseudomonas aerguinosa O10	L8643				
**65**	Pseudomonas aerguinosa O10 10a,10b	IATS 10, OPS				
**66**	Pseudomonas aerguinosa O10 10a,10c	IATS 19, OPS				
**67**	Pseudomonas aerguinosa O11 11a,11b	IATS 11, OPS				
**68**	Pseudomonas aerguinosa O12 12	IATS 12, OPS Habs 12				
**6**	Pseudomonas aerguinosa O13 (Sandvik serotype II)	PO13 Core-linked				
**69**	Pseudomonas aerguinosa O13 13a,13c	IATS 14, OPS				
**70**	Pseudomonas aerguinosa O14 14	IATS 17,OPS Meitert X				
**71**	Pseudomonas aerguinosa O15 15	IATS 15, OPS				
**50**	Pseudomonas aerguinosa O2 (2a),2d,2f	IATS 10 , OPS				
**5**	Pseudomonas aerguinosa O2 (Fisher immunotype 3)	PO2 Core-linked				
**51**	Pseudomonas aerguinosa O2 2a,2b	IATS 16 OPS				
**52**	Pseudomonas aerguinosa O2 2a,2b,2e	IATS NO, OPS		11.76		
**53**	Pseudomonas aerguinosa O2 2a,2d	IATS 5 OPS				
**54**	Pseudomonas aerguinosa O2 Immuno 7	IATS 18, OPS	5.2			
**55**	Pseudomonas aerguinosa O3 3a,3b	IATS NO, OPS				
**56**	Pseudomonas aerguinosa O3 3a,3b,3c	IATS 3, OPS	3.4			
**57**	Pseudomonas aerguinosa O3 3a,3d	IATS NO, OPS				
**3**	Pseudomonas aerguinosa O4 (Habs serotype 4)	PO4 Core-linked				
**58**	Pseudomonas aerguinosa O4 4a,4c	IATS NO, OPS				
**59**	Pseudomonas aerguinosa O6 6a	IATS 6, OPS				
**60**	Pseudomonas aerguinosa O6 6a,6c	IATS NO, OPS				
**61**	Pseudomonas aerguinosa O6 Immuno 1	IATS NO, OPS				
**8**	Pseudomonas aerguinosa O6a (Habs serotype6, fraction IIa)	PO6a Core-linked-O-unit				
**9**	Pseudomonas aerguinosa O6a (Habs serotype6, fraction IIb)	PO6a unsubstituted core				
**62**	Pseudomonas aerguinosa O7 7a,7b,7c	IATS 7,LPS				
**63**	Pseudomonas aerguinosa O7 7a,7b,7d	IATS 8,LPS				
**64**	Pseudomonas aerguinosa O7 7a,7d	IATS NO, LPS				
**7**	Pseudomonas aerguinosa O9 (9a, 9b, 9d)	PO9 Core-linked		7.57		
**20**	Salmonella enterica abortus equi LPS	L5886				
**299**	Salmonella enterica O17	PS				
**300**	Salmonella enterica O28	PS				
**301**	Salmonella enterica O47	PS				
**302**	Salmonella enterica O55	PS				
**15**	Salmonella enteritidis dodeca saccharide	1262				
**22**	Salmonella enteritidis LPS	L2012				
**14**	Salmonella typhimurium dodeca saccharide	4809				
**16**	Salmonella typhimurium LPS	L2262				
**10**	Salmonella typhimurium SL 11881 (Re mut)	LPS-L9516				
**12**	Salmonella typhimurium SL 684 (Rc mut)	LPS-L5891				
**11**	Salmonella typhimurium TV 119 (Ra mut)	LPS-L6016				
**21**	Salmonella typhosa LPS	L2387				
**17**	Serratia marcescens LPS	L6136				
**25**	Shigella bodyii type10					
**23**	Shigella bodyii type2			5.72		
**24**	Shigella bodyii type4					
**195**	Shigella boydii type 1	LPSOH		4.46		
**199**	Shigella boydii type 11	OPS		4.48		
**200**	Shigella boydii type 12	OPS				
**292**	Shigella boydii type 13	LPSOH				
**293**	Shigella boydii type 14	LPSOH				
**201**	Shigella boydii type 15	OPS				
**202**	Shigella boydii type 16	OPS				
**203**	Shigella boydii type 17	OPS				
**204**	Shigella boydii type 18	OPS				
**196**	Shigella boydii type 3	OPS				
**197**	Shigella boydii type 5	OPS				
**289**	Shigella boydii type 6	PS				
**290**	Shigella boydii type 7	PS				
**291**	Shigella boydii type 8	PS				
**198**	Shigella boydii type 9	OPS				
**28**	Shigella dysennteriae type 11					
**29**	Shigella dysennteriae type 13					
**26**	Shigella dysennteriae type 3					
**27**	Shigella dysennteriae type 8 (batch 12)					
**288**	Shigella dysenteriae type 1	PS				
**215**	Shigella dysenteriae type 2	LPSOH				
**216**	Shigella dysenteriae type 4	OPS				
**217**	Shigella dysenteriae type 5	OPS				
**218**	Shigella dysenteriae type 6 SR-strain	SR-strain				
**219**	Shigella dysenteriae type 7	OPS				

**220**	Shigella dysenteriae type 8 (Russian)	OPS				
**221**	Shigella dysenteriae type 9	OPS				
**276**	Shigella flexneri type 1a	PS		11.26		
**277**	Shigella flexneri type 1b	PS				
**278**	Shigella flexneri type 2a	PS	6.3			
**279**	Shigella flexneri type 2b	PS				
**280**	Shigella flexneri type 3a	PS				
**281**	Shigella flexneri type 3b	PS				
**282**	Shigella flexneri type 4a	PS				
**283**	Shigella flexneri type 4b	PS				
**284**	Shigella flexneri type 5b	PS				
**286**	Shigella flexneri type 6	PS				
**285**	Shigella flexneri type 6a	PS				
**287**	Shigella flexneri type X	PS				
**226**	Streptococcus pneumoniae type 1 (Danish type 1)	161-X // Capsular PS	3.4			
**233**	Streptococcus pneumoniae type 12 (Danish type 12F)	193-X// Capsular PS				
**234**	Streptococcus pneumoniae type 14 (Danish type 14)	197-X// Capsular PS	2.0			100.0
**235**	Streptococcus pneumoniae type 17 (Danish type 17F)	201-X// Capsular PS				
**236**	Streptococcus pneumoniae type 19 (Danish type 19F)	205-X// Capsular PS		8.44		
**227**	Streptococcus pneumoniae type 2 (Danish type 2)	165-X// Capsular PS				
**237**	Streptococcus pneumoniae type 20 (Danish type 20)	209-X// Capsular PS				
**238**	Streptococcus pneumoniae type 22 (Danish type 22F)	213-X// Capsular PS	5.5			
**239**	Streptococcus pneumoniae type 23 (Danish type 23F)	217-X// Capsular PS				
**240**	Streptococcus pneumoniae type 26 (Danish type 6B)	225-X// Capsular PS		19.05		
**228**	Streptococcus pneumoniae type 3 (Danish type 3)	169-X// Capsular PS	3.5			
**241**	Streptococcus pneumoniae type 34 (Danish type 10A)	229-X// Capsular PS				
**229**	Streptococcus pneumoniae type 4 (Danish type 4)	173-X// Capsular PS				
**242**	Streptococcus pneumoniae type 43 (Danish type 11A)	233-X// Capsular PS	0.2		2.0	
**230**	Streptococcus pneumoniae type 5 (Danish type 5)	177-X// Capsular PS		9.66		
**243**	Streptococcus pneumoniae type 51 (Danish type 7F)	237-X// Capsular PS				
**244**	Streptococcus pneumoniae type 54 (Danish type 15B)	241-X// Capsular PS	2.6			69.68
**245**	Streptococcus pneumoniae type 56 (Danish type 18C)	245-X// Capsular PS				
**246**	Streptococcus pneumoniae type 57 (Danish type 19A)	249-X// Capsular PS				
**247**	Streptococcus pneumoniae type 68 (Danish type 9V)	253-X// Capsular PS				
**248**	Streptococcus pneumoniae type 70 (Danish type 33F)	257-X// Capsular PS	7.7			52.77
**231**	Streptococcus pneumoniae type 8 (Danish type 8)	185-X// Capsular PS				
**232**	Streptococcus pneumoniae type 9 (Danish type 9N)	189-X// Capsular PS				
**309**	Yeast Mannan					
**312**	Yeast Mannan Sigma M-3640					
**153**	Yersinia pestis 1146-25	Core oligo saccharide				
**177**	Yersinia pestis 260(11)-37C-186	LPS				
**178**	Yersinia pestis 260(11)-37C-187	LPS				
**179**	Yersinia pestis 260(11)-37C-416	LPS				
**180**	Yersinia pestis 260(11)-37C-417	LPS				
**183**	Yersinia pestis I-2377-25C	OS				
**184**	Yersinia pestis I-2377-37C	LPS				
**249**	Yersinia pestis KM218-6C	OS				
**176**	Yersinia pestis KM260(11)-6C	LPS				
**252**	Yersinia pestis KM260(11)-wabC/waaL	OS				
**251**	Yersinia pestis KM260(11)-wabD/waaL	OS				
**250**	Yersinia pestis KM260(11)-yjhW-6C	OS				
**181**	Yersinia pestis P-1680-25C	OS				
**182**	Yersinia pestis P-1680-37C	LPS				
**156**	Yersinia pestis, 0KM218-37	LPS				
**152**	Yersinia pestis, 1146-25	LPS				
**154**	Yersinia pestis, 1146-37	LPS				
**155**	Yersinia pestis, 1146-37	Core oligo saccharide				
**172**	Yersinia pestis, 11M-25	LPS				
**173**	Yersinia pestis, 11M-37	LPS				
**171**	Yersinia pestis, KIMD1-25	Core oligo saccharide				
**170**	Yersinia pestis, KIMD1-37	Core oligo saccharide				
**158**	Yersinia pestis, KM218-25	LPS				
**159**	Yersinia pestis, KM218-25	Core oligo saccharide				
**157**	Yersinia pestis, KM218-37	Core oligo saccharide				
**162**	Yersinia pestis, KM260(11)-Δ0186	LPS				
**163**	Yersinia pestis, KM260(11)-Δ0186	Core oligo saccharide				
**148**	Yersinia pestis, KM260(11)-Δ0187	LPS				
**149**	Yersinia pestis, KM260(11)-Δ0187	Core oligo saccharide				
**160**	Yersinia pestis, KM260(11)-ΔpmrF	LPS				
**161**	Yersinia pestis, KM260(11)-ΔpmrF	Core oligo saccharide				
**150**	Yersinia pestis, KM260(11)-Δrfe	LPS				
**151**	Yersinia pestis, KM260(11)-Δrfe	Core oligo saccharide				
**166**	Yersinia pestis, KM260(11)-ΔwaaL	LPS				
**164**	Yersinia pestis, KM260(11)-ΔwaaQ	LPS				
**165**	Yersinia pestis, KM260(11)-ΔwaaQ	Core oligo saccharide				
**167**	Yersinia pestis, KM260(11)-25	LPS				

**168**	Yersinia pestis, KM260(11)-25	Core oligo saccharide				
**169**	Yersinia pestis, KM260(11)-37	Core oligo saccharide				
**254**	Yersinia pseudotuberculosis 85pCad-20C	OS				
**253**	Yersinia pseudotuberculosis 85pCad-37C	OS				
**255**	Yersinia pseudotuberculosis O:2a	PS				
**256**	Yersinia pseudotuberculosis O:2a-dhmA	PS				
**257**	Yersinia pseudotuberculosis O:2c	PS				
**258**	Yersinia pseudotuberculosis O:3	PS				
**259**	Yersinia pseudotuberculosis O:4b	PS				

Supplementary Table 2. Gal-3 and Gal-3C binding to glycans present on the microbial glycan microarrays. Each glycan is numbered, with each structure listed and the apparent K_D value or % max binding at the highest concentration tested for glycan binding that did not result in saturation over the concentrations tested. Gal-3: galectin-3. Gal-3C: the C-terminal domain of galectin-3.
